# N6-Methyladenosine-Related Long Non-coding RNA Signature Associated With Prognosis and Immunotherapeutic Efficacy of Clear-Cell Renal Cell Carcinoma

**DOI:** 10.3389/fgene.2021.726369

**Published:** 2021-10-15

**Authors:** Tianming Ma, Xiaonan Wang, Jiawen Wang, Xiaodong Liu, Shicong Lai, Wei Zhang, Lingfeng Meng, Zijian Tian, Yaoguang Zhang

**Affiliations:** ^1^ Department of Urology, Beijing Hospital, National Center of Gerontology, Institute of Geriatric Medicine, Chinese Academy of Medical Sciences, Beijing, China; ^2^ Graduate School of Peking Union Medical College, Chinese Academy of Medical Sciences, Beijing, China; ^3^ Department of Radiology, Beijing Hospital, National Center of Gerontology, Institute of Geriatric Medicine, Chinese Academy of Medical Sciences, Beijing, China

**Keywords:** clear-cell renal cell carcinoma, N6-methyladenosine, lncRNA, prognostic signature, immune infiltration

## Abstract

Increasing evidence suggests that N6-methyladenosine (m6A) and long non-coding RNAs (lncRNAs) play important roles in cancer progression and immunotherapeutic efficacy in clear-cell renal cell carcinoma (ccRCC). In this study, we conducted a comprehensive ccRCC RNA-seq analysis using The Cancer Genome Atlas data to establish an m6A-related lncRNA prognostic signature (m6A-RLPS) for ccRCC. Forty-four prognostic m6A-related lncRNAs (m6A-RLs) were screened using Pearson correlation analysis (|R| > 0.7, *p* < 0.001) and univariable Cox regression analysis (*p* < 0.01). Using consensus clustering, the patients were divided into two clusters with different overall survival (OS) rates and immune status according to the differential expression of the lncRNAs. Gene set enrichment analysis corroborated that the clusters were enriched in immune-related activities. Twelve prognostic m6A-RLs were selected and used to construct the m6A-RLPS through least absolute shrinkage and selection operator Cox regression. We validated the differential expression of the 12 lncRNAs between tumor and non-cancerous samples, and the expression levels of four m6A-RLs were further validated using Gene Expression Omnibus data and Lnc2Cancer 3.0 database. The m6A-RLPS was verified to be an independent and robust predictor of ccRCC prognosis using univariable and multivariable Cox regression analyses. A nomogram based on age, tumor grade, clinical stage, and m6A-RLPS was generated and showed high accuracy and reliability at predicting the OS of patients with ccRCC. The prognostic signature was found to be strongly correlated to tumor-infiltrating immune cells and immune checkpoint expression. In conclusion, we established a novel m6A-RLPS with a favorable prognostic value for patients with ccRCC. The 12 m6A-RLs included in the signature may provide new insights into the tumorigenesis and allow the prediction of the treatment response of ccRCC.

## Introduction

Kidney cancer, of which renal cell carcinoma (RCC) is the major histological type, is a common type of cancer with a high mortality rate and steadily rising morbidity rate worldwide (Bray et al., 2018). In 2020, there were approximately 73,750 new cases of kidney cancer and approximately 14,830 deaths due to kidney cancer in United States (Siegel et al., 2020). Clear-cell (cc)RCC is the most common subtype of RCC, accounting for more than 70% of cases (Rini et al., 2009). Although surgery is an effective treatment for early-stage ccRCC, relapse or metastasis after curative treatment occurs in 30% of cases. Unfortunately, the prognosis for patients with metastasis is generally dismal because ccRCC is insensitive to conventional radiotherapy and chemotherapy, even to tyrosine kinase inhibitors (TKIs) and immune-checkpoint inhibitors (ICIs) (Ljungberg et al., 2019; Mori et al., 2021). Therefore, it is imperative to elucidate the mechanisms underlying ccRCC development to identify effective treatments.

N6-methyladenosine (m6A), a reversible and abundant modification on mRNAs and non-coding RNAs, has been demonstrated to affect various aspects of RNA metabolism, including splicing, stability, nuclear export, and translation (Huang et al., 2020). Several studies support the hypothesis that aberrant expression of m6A regulators, i.e., “writers” (methyltransferases), “readers” (binding proteins), and “erasers” (demethylases), potentially participates in carcinogenesis, cancer development, or tumor-suppressive activities in various types of cancer, including ccRCC (Wang Q. et al., 2020; Huang et al., 2020). For instance, high expression of methyltransferase 3 (METTL3), an m6A methyltransferase, reportedly promotes the progression of gastric cancer, lung cancer, and prostate cancer (Cai et al., 2019; Wang T. et al., 2020; Wanna-Udom et al., 2020). In contrast, downregulation of METTL3 in endometrial cancer and decreased the expression of alkB homolog 5 (ALKBH5) and fat mass and obesity-associated protein (FTO), which are both “erasers,” in ccRCC, has been suggested to be related to tumorigenicity and dismal prognosis (Liu et al., 2018; Strick et al., 2020).

Long non-coding RNAs (lncRNAs) also actively regulate various biological processes, including tumorigenesis, proliferation, and immunity (Peng et al., 2017; Taniue and Akimitsu, 2021). In ccRCC, lncRNA SNHG1 modulates immune escape by targeting miR-129-3p to activate STAT3 and PD-L1 (Tian et al., 2021), and LINC00641 facilitates ccRCC progression by sponging miR-340-5p (Zhang et al., 2021). Abundant evidence supports that m6A modifications and lncRNAs interactively affect the growth and development of various cancers (Wang X. et al., 2020; Huang et al., 2020; Yang et al., 2020). However, few studies have investigated the exact mechanism by which m6A modifications are involved in lncRNA-dependent ccRCC progression and prognosis. Recently, several prognostic signatures for ccRCC based on m6A regulators or lncRNAs alone have been identified (Zeng et al., 2019; Chen et al., 2020). However, to the best of our knowledge, an accurate and applicable prognostic signature based on m6A-related lncRNAs for patients with ccRCC has not been formulated. Therefore, we aimed to investigate the prognostic significance of m6A-related lncRNAs (m6A-RLs), and to develop an m6A-related lncRNA prognostic signature (m6A-RLPS) to predict the survival outcomes in patients with ccRCC.

## Materials and Methods

### Data Sources

We collected transcriptome RNA-sequencing (RNA-seq) data of 539 ccRCC samples and 72 adjacent non-tumor samples with corresponding clinical data from the Cancer Genome Atlas (TCGA) database. Detailed clinicopathological information of the patients is provided in Supplementary Table S1. Patients with incomplete information were excluded. Expression matrices of 23 m6A-related genes (*METTL3*, *METTL14*, *METTL16*, *WTAPI*, *VIRMA*, *ZC3H13*, *RBM15*, *RBM15B*, *YTHDC1*, *YTHDC2*, *YTHDF1*, *YTHDF2*, *YTHDF3*, *HNRNPC*, *FMR1*, *LRPPRC*, *HNRNPA2B1*, *IGFBP1*, *IGFBP2*, *IGFBP3*, *RBMX*, *FTO*, and *ALKBH5*) were also obtained according to recent publications (Deng et al., 2018; Chen et al., 2019). To validate the expression of target lncRNAs, we extracted gene expression profile data from four datasets with more than 60 ccRCC samples in the Gene Expression Omnibus (GEO) database, including GSE17895 (tumor: *n* = 151, normal: *n* = 9), GSE40435 (tumor: *n* = 101, normal: *n* = 101), GSE46699 (tumor: *n* = 67, normal: *n* = 63), and GSE53757 (tumor: *n* = 72, normal: *n* = 72) (Dalgliesh et al., 2010; Wozniak et al., 2013; Eckel-Passow et al., 2014; von Roemeling et al., 2014). In addition, an updated Lnc2Cancer 3.0 (http://bio-bigdata.hrbmu.edu.cn/lnc2cancer) database, which hosts comprehensive data on experimentally supported lncRNAs and circular RNAs associated with human cancers (Gao et al., 2021), was used to analyze lncRNA expression further.

### m6A-Related lncRNA Identification

The lncRNA profile obtained from TCGA was first screened against the human reference genome (GRCh38. p12; https://www.ncbi.nlm.nih.gov/genome). Pearson correlation analysis was used to identify m6A-RLs (|Pearson’s R| > 0.7, *p* < 0.001). Then, univariable Cox regression analysis was performed to determine prognostic m6A-RLs related to the overall survival (OS) (*p* < 0.01). The Wilcoxon rank-sum test was utilized to compare the expression levels (visualized using heatmaps) of the prognostic m6A-RLs between tumor and normal tissues.

### Consensus Clustering Analysis

To explore the expression features of prognostic m6A-RLs further, we clustered the ccRCC samples into different groups according to the differential expression of the lncRNAs using the ConsensusClusterPlus R package (Chen et al., 2020). Survival analysis and the chi-square test or Fisher’s exact test were used to compare the survival rates between the clusters and to determine the relationships between the clinicopathological parameters and the clusters. Heatmaps were created using the “pheatmap” R package to visualize differential expression of the m6A-RLs and clinicopathological parameters in the different groups (Li, 2012).

### Gene Set Enrichment Analysis and Tumor-Infiltrating Immune Cell Profiling

GSEA was conducted using the Hallmark, C2 KEGG v.7.1, C5 GO, and C7 v.6.2 gene sets in GSEA v.4.0.3 (http://www.broadinstitute.org/gsea) to investigate potential m6A-RL pathways and functions. The ESTIMATE algorithm was used to determine immune, stromal, and ESTIMATE scores for each sample. These scores represent the ratios of immune and stromal components and the total proportion of these components in the tumor microenvironment (TME) (Yoshihara et al., 2013). The CIBERSORT algorithm was used to estimate the abundance of 22 tumor-infiltrating immune cells (TICs) (Chen et al., 2018). Only tumor samples with *p* < 0.05 were retained for subsequent analysis.

### Analysis of the Associations Between Clusters and Tumor-Infiltrating Immune Cells

The Wilcoxon rank-sum test was used to determine the relationships between TICs in the different clusters. Some common immune checkpoints, including CTLA-4, LAG-3, HAVCR2 (TIM-3), PDCD1 (PD-1), and TIGHT, were screened out to compare differences in expression between tumor and normal tissues and in the differential clusters, as well as to investigate the correlations between the expression levels of the genes and hub m6A-RLs.

### Establishment and Validation of the m6A-Related lncRNA Prognostic Signature

The entire cohort was randomly divided into training and first validation groups at a 1:1 ratio. Thereafter, all the samples were randomly divided into the second and third validation cohorts at a ratio of 3:7. The training cohort was used to construct the m6A-RLPS to predict the prognosis of ccRCC patients. The selected m6A-related candidate lncRNAs mentioned above were subjected to least absolute shrinkage and selection operator (LASSO) Cox regression analysis for establishing an m6A-RLPS for ccRCC (Friedman et al., 2010). A risk score was then calculated for each patient using the following formula: risk score = $\sum_{i=1}^{n}Coef_{i}*Expr_{i}$ , where Coef_i_ is the coefficient and Expr_i_ is the expression value of the *i*th lncRNA in the signature. Univariable Cox regression and Kaplan–Meier survival analyses were used to analyze the prognostic significance of the selected lncRNAs and their co-expressed m6A genes. The Wilcoxon rank-sum test was used to investigate differential expression of the selected lncRNAs and co-expressed m6A genes between tumor and non-cancerous samples in TCGA, which was further validated using GEO datasets and Lnc2Cancer 3.0.

Next, patients were classified into low- and high-risk groups according to the median risk score. The OS and disease-specific survival (DSS) were compared between the groups based on Kaplan–Meier curves. Time-dependent receiver operating-characteristic (ROC) curves with area under curve (AUC) values for the 1-, 3-, and 5-years OS and DSS rates were used to estimate the prognostic prediction efficacy of the m6A-RLPS. Heatmaps were generated to visualize the differential expression of the prognostic m6A-RLs in the low- and high-risk groups. Univariable and multivariable Cox regression analyses were used to investigate the independent predictive value of the risk score and clinicopathological parameters for the survival of patients with ccRCC. Survival analysis was conducted to further elucidate the prognostic ability of the risk score in subgroups stratified by age, sex, grade, clinical stage, and T stage. A nomogram was built based on the multivariate Cox regression analysis results and was comprehensively evaluated using the concordance index (C-index) and calibration curves.

### Correlation Between the m6A-Related lncRNA Prognostic Signature and Clinicopathological Parameters

Associations between the clinicopathological parameters, immune score, clusters, and m6A-RLPS risk levels were assessed using the chi-square test or Fisher’s exact test (shown in heatmaps). Student’s *t*-test was used to determine the relationships between the risk scores and clinicopathological parameters, including age, sex, grade, clinical stage, T stage, cluster, immune score, stromal score, and ESTIMATE score. Additionally, survival analysis was employed to explore associations between all these factors and patient OS.

### Correlation Between the m6A-Related lncRNA Prognostic Signature and Immune-Related Features

Correlations between the m6A-RLPS and immune cells were evaluated using the Wilcoxon rank-sum test and Spearman correlation analysis. In addition, we downloaded data of different cancer cell lines from the NCI-60 database (https://discover.nci.nih.gov/cellminer/home.do). Thereafter, the m6A-RLPS was also comprehensively analyzed to determine its relationship with the expression of some immune checkpoint proteins and drug sensitivity of some TKIs involved in ccRCC.

### Statistical Analysis

All analyses were performed using R v.4.0.3 (http://www.R-project.org). The Wilcoxon rank-sum test was used to investigate differential expression of lncRNAs and TICs. The Pearson correlation analysis was performed to identify m6A-RLs. Kaplan-Meier method and log rank test were used for comparing the OS and DSS between various groups, incorporating the high and low-risk groups and other subgroups based on the expression of each of the 12 m6A-RLs. Student’s *t*-test was used to determine the relationships between the risk scores and clinicopathological parameter. Chi-square test or Fisher’s exact test was used to analyze associations between the clinicopathological parameters, immune score, clusters, and m6A-RLPS. Univariable and multivariable Cox regression analyses were applied to investigate the independent predictive value of the m6A-RLPS for the OS and DSS of patients with ccRCC. Statistical significance was set at *p* < 0.05 (two-tailed).

## Results

### Identification of Prognostic m6A-RLs in Clear-Cell Renal Cell Carcinoma

**FIGURE 1:**
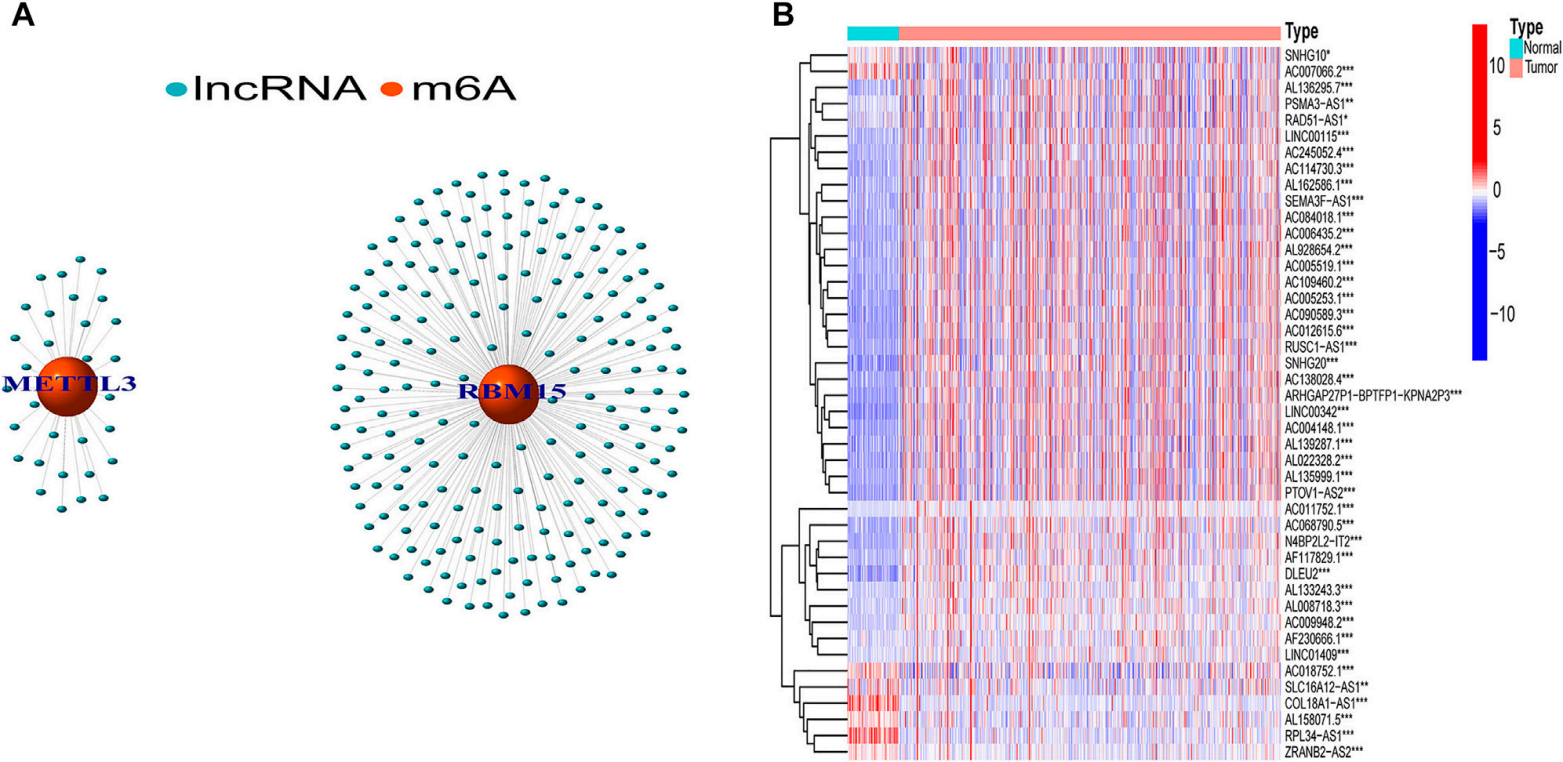
Screening of hub lncRNAs. **(A)** Network of the 23 selected m6A-related genes and their associated lncRNAs. **(B)** Differential expression of the 44 prognostic m6A-RLs in tumor and normal samples. *p < 0.05, **p < 0.01, ***p < 0.001.

In total, 239 m6A-related lncRNAs were found to be significantly correlated with 23 m6A-related genes using Pearson correlation analysis (|R| > 0.7, *p* < 0.001, Figure 1A, Supplementary Table S2). After excluding patients without cancer or survival data, we merged the survival data with lncRNA expression data of each patient (final patient number = 530). Then, we identified 44 lncRNAs related to prognosis (*p* < 0.01, Supplementary Table S3) using univariable Cox regression analysis. As shown in Figure 1B, the expression of these prognostic m6A-RLs differed significantly between normal and ccRCC tissues.

### Consensus Clustering of m6A-Related lncRNAs Identified in Molecular Subtypes Clear-Cell Renal Cell Carcinoma

**FIGURE 2:**
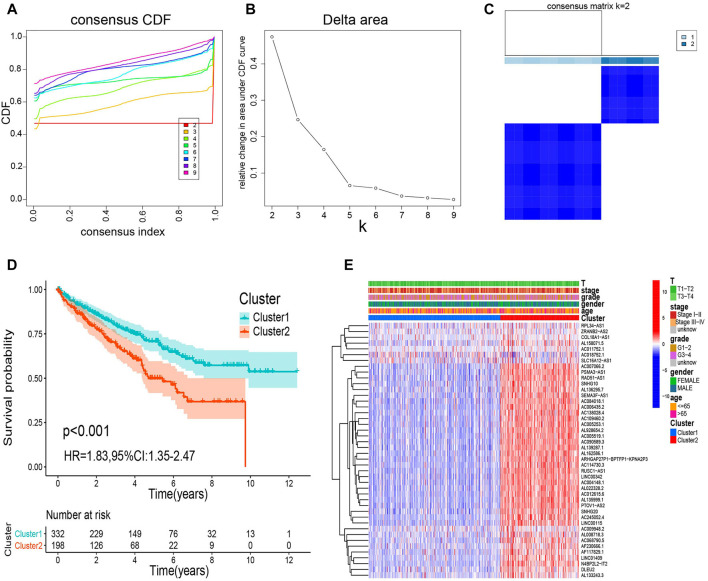
Consensus clustering analysis of m6A-RL expression. **(A)** Consensus clustering cumulative distribution function for k = 2 to 9. **(B)** Relative change in the area under the cumulative distribution function curve for k = 2 to 9. **(C)** Consensus matrix for k = 2. **(D)** Kaplan–Meier survival analysis of the overall survival (OS) for the two clusters. **(E)** Relationships between m6A-RL expression and clinicopathological parameters.

We categorized all samples into groups according to the expression profiles of the prognostic m6A-RLs, using a consensus clustering algorithm. We found that k = 2 was the most suitable choice to sort the entire cohort into two groups: cluster 1 (*n* = 332) and cluster 2 (*n* = 198) (Figures 2A–C). Kaplan–Meier curves revealed that patients in cluster 2 had a worse OS than those in cluster 1 (*p* < 0.001, Figure 2D). Nevertheless, as shown in Figure 2E, there were no distinct differences in clinical variables (age, sex, grade, clinical stage, and T stage) between the clusters. Thus, the consensus clustering results were clearly associated with the survival of patients with ccRCC.

### GSEA and Immune-Related Analysis of the Two Clusters

**FIGURE 3:**
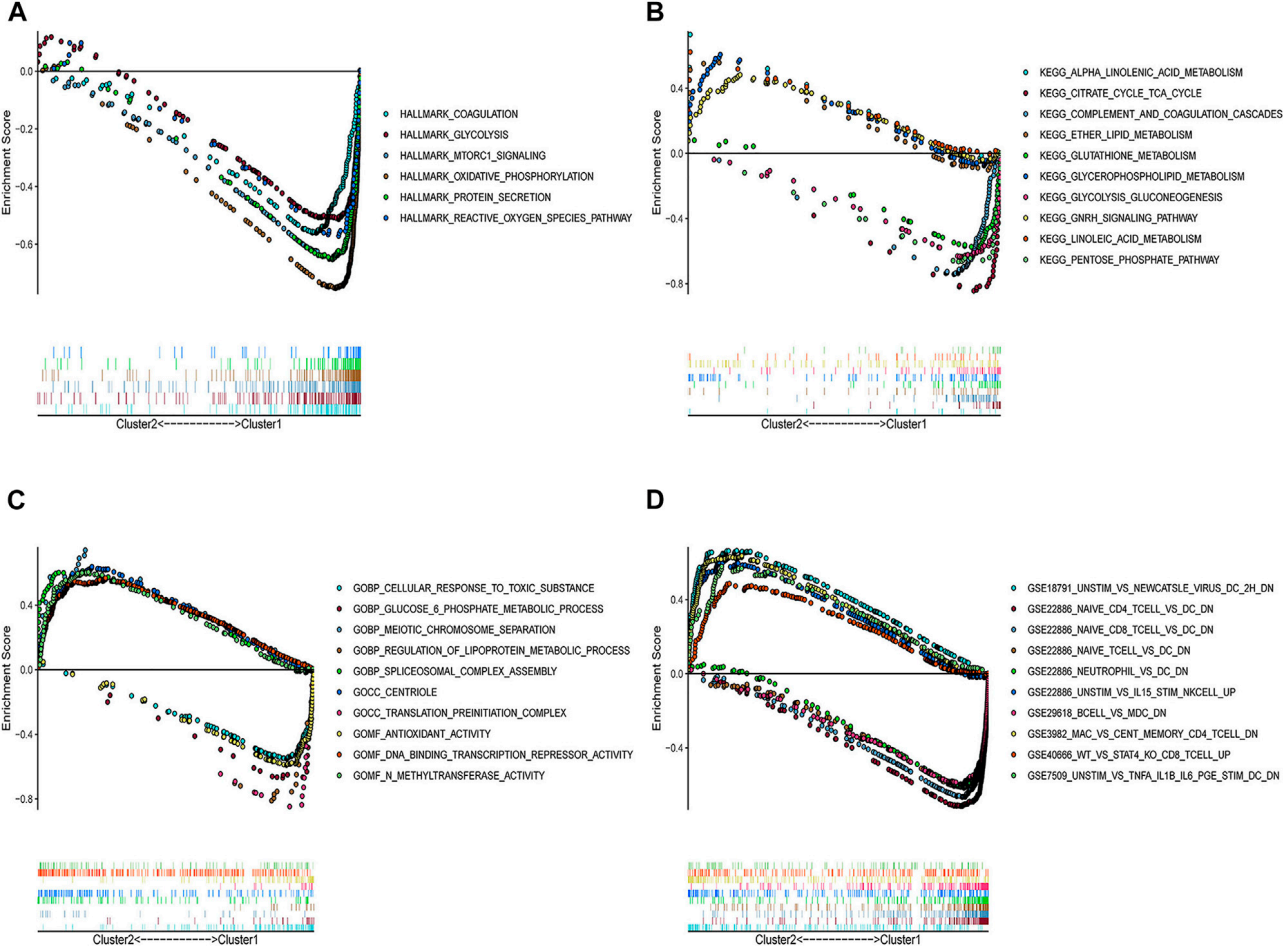
GSEA for the two clusters. **(A)** Tumor hallmarks were enriched in cluster 1. Multi-GSEA enrichment curves for the **(B)** C2 KEGG collection, **(C)** C5 GO collection, and **(D)** C7 collection for two clusters. All the | NES | > 1, Nominal *p* < 0.05, and false discovery rate-corrected *q* < 0.25. NES: Normalized enrichment score.

To determine the potential pathways and functions associated with the prognostic m6A-RLs in ccRCC, GSEA was applied to the two clusters. Notably, several tumor hallmarks, such as coagulation, glycolysis, and mTORC1 signaling, were predominantly enriched in cluster 1 (nominal *p* < 0.05 and false discovery rate-corrected *q* < 0.05, Figure 3A). C2 Kyoto Encyclopedia of Genes and Genomes (KEGG) and C5 Gene Ontology (GO) analyses similarly revealed multiple tumor-related signaling pathways in the samples (Figures 3B,C). Notably, several enriched immune-related signaling pathways and genes were identified through both C2 KEGG and C5 GO analyses. C7 collection analysis indicated that multiple immune-functional gene sets were enriched in clusters 1 and 2 (Figure 3D).

**FIGURE 4:**
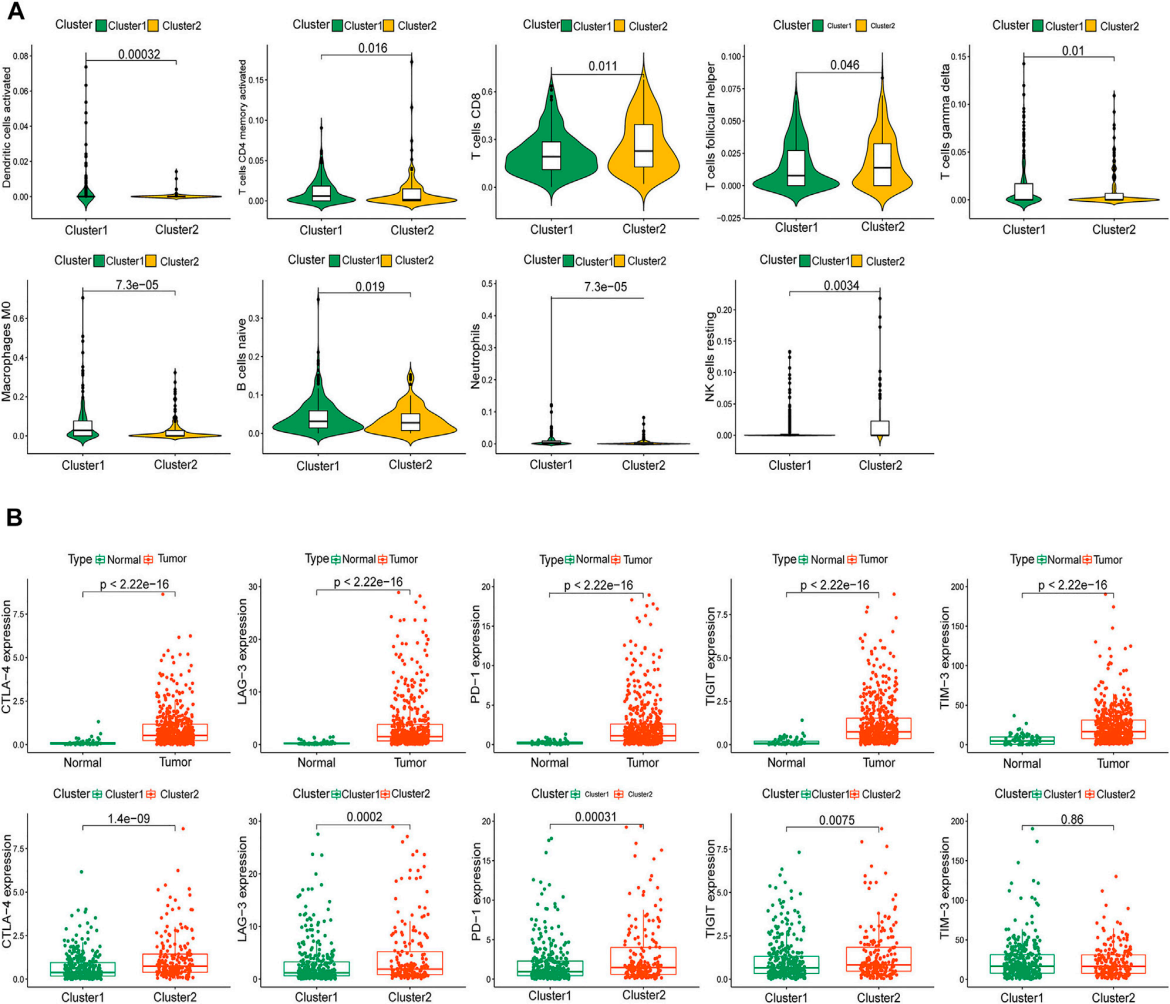
Immune status of patients in the two clusters. **(A)** Identification of nine types of tumor-infiltrating immune cells (TICs) in the two clusters with *p* < 0.05. **(B)** Distribution of five immune checkpoints.

Additionally, we thoroughly evaluated the correlations between the two clusters and TICs in the ccRCC samples. Activated dendritic cells, activated memory CD4 T cells, CD8 T cells, follicular helper T cells, gamma delta T cells, M0 macrophages, naive B cells, neutrophils, and resting natural killer cells were significantly associated with the clusters (Figure 4A). Given the potential immunomodulatory effects of the expression of the m6A-RLs, we determined the correlations between the clusters and some immune checkpoints. Notably, all five immune checkpoints evaluated, except TIM-3, were highly expressed in tumor samples and cluster 2 (Figure 4B). The correlations between the 44 lncRNAs and the five immune checkpoints are shown in Supplementary Figure S1, and most of these correlations were significant. Based on these results, we speculated that the poor prognosis of patients in cluster 2 was probably due to the upregulation of the immune checkpoints.

### Construction and Validation of the m6A-Related lncRNA Prognostic Signature

**FIGURE 5:**
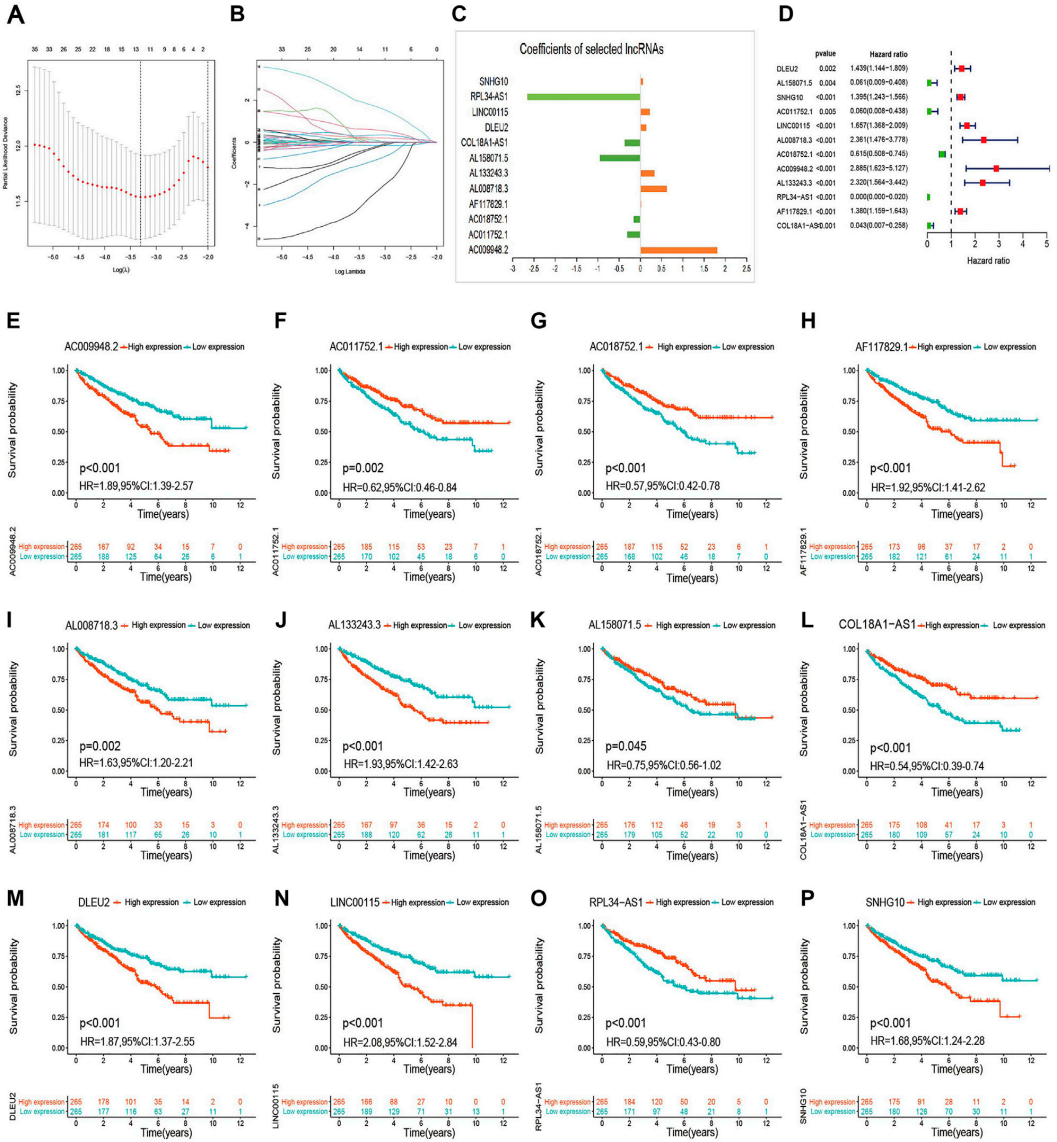
Establishment of the m6A-RLPS. **(A–C)** LASSO Cox regression analysis to determine the 12 m6A-RLs and their corresponding coefficients. **(D)** Forest plot of the 12 m6A-RLs. **(E–P)** Kaplan–Meier curves indicating different overall survival (OS) of patients with different expression levels of the 12 m6A-RLs.

The entire cohort with complete survival information was randomly divided into training (*n* = 266) and the first validation (*n* = 264) cohort at a ratio of 1:1 and was then divided into the second (*n* = 156) and third (*n* = 374) validation cohorts at a ratio of 3:7. The risk model was developed using the training cohort and validated using the validation cohorts. We conducted LASSO Cox regression analysis to screen the 44 prognostic m6A-RLs and the 12 lncRNAs incorporated into the m6A-RLPS for lncRNAs that would allow prediction of the OS of patients with ccRCC (Figures 5A–C). The risk score for each patient was calculated as follows: risk score = (1.083 × AC009948.2 expression) + (−0.303 × AC011752.1 expression) + (−0.148 × AC018752.1 expression) + (−0.002 × AF117829.1 expression) + (0.616 × AL008718.3 expression) + (0.334 × AL133243.3 expression) + (−0.943 × AL158071.5 expression) + (−0.363 × COL18A1-AS1 expression) + (0.141 × DLEU2 expression) + (0.217 × LINC00115 expression) + (−2.645 × RPL34-AS1 expression) + (0.053 × SNHG10 expression) (expression refers to the normalized log levels of each lncRNA). Both univariable Cox regression analysis and Kaplan–Meier log-rank test-based survival analysis supported the remarkable prognostic significance of all 12 lncRNAs, among which AC011752.1, AC018752.1, AL158071.5, COL18A1-AS1, and RPL34-AS1 were protective factors with a hazard ratio < 1, whereas all others were risk factors (Figure 5D–P).

**FIGURE 6:**
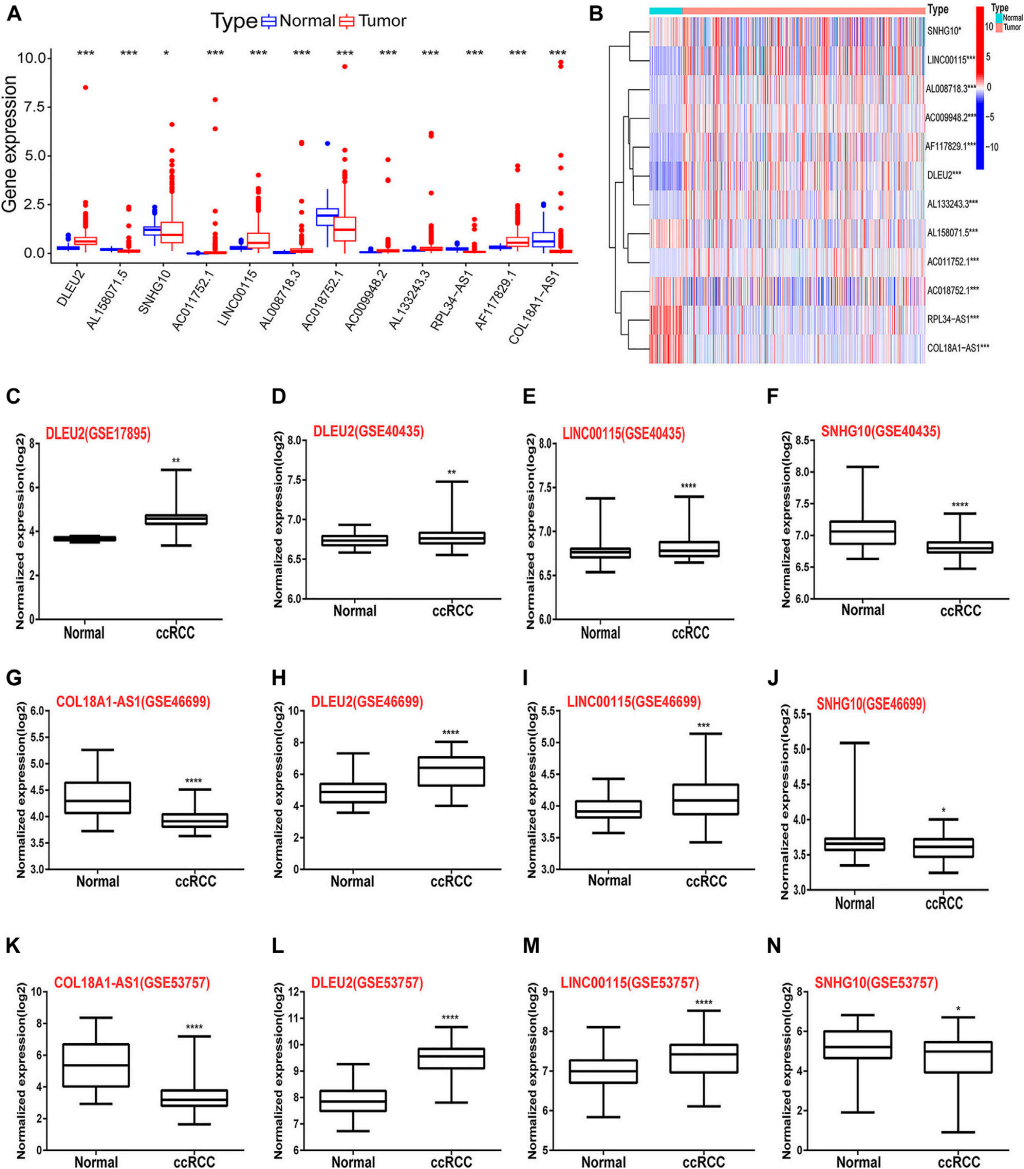
Differential expression of the 12 m6A-RLs between tumor and normal renal tissues. **(A)** Boxplots and **(B)** heatmap of the expression of the 12 m6A-RLs based on TCGA data. **(C–N)** Verification of the differential expression of several lncRNAs among the 12 m6A-RLs based on the GEO data. *p < 0.05; **p < 0.01; ***p < 0.001, ****p < 0.0001.

The relative expression levels of the 12 signature lncRNAs differed significantly between normal and ccRCC tissues based on TCGA data (Figures 6A,B). In addition, we collected the expression data from the GEO DataSets platform for validation analysis of the 12 lncRNAs. Ultimately, only GSE17895 (contained DLEU2), GSE40435 (contained DLEU2, LINC00115, and SNHG10), GSE46699 and GSE53757 (both contained COL18A1-AS1, DLEU2, LINC00115, and SNHG10) were included in the subsequent analyses. The results showed that DLEU2 and LINC00115 were markedly overexpressed in ccRCC samples compared with normal tissues, whereas the expression levels of COL18A1-AS1 and SNHG10 were remarkably lower in tumor tissues than those in the adjacent non-tumor samples (Figure 6C–N). Furthermore, Wilcoxon rank-sum test in Lnc2Cancer 3.0 showed the similar differential expression of COL18A1-AS1, DLEU2, LINC00115, RPL34-AS1, and SNHG10 (Supplementary Figure S2). These findings were in accordance with the results of TCGA analysis. Moreover, the co-expression of m6A genes (*METTL3* and RNA binding motif protein 15 (*RBM15*)) of the 12 lncRNAs also differed between tumor and normal tissues and was associated with OS in ccRCC (Supplementary Figure S3).

**FIGURE 7:**
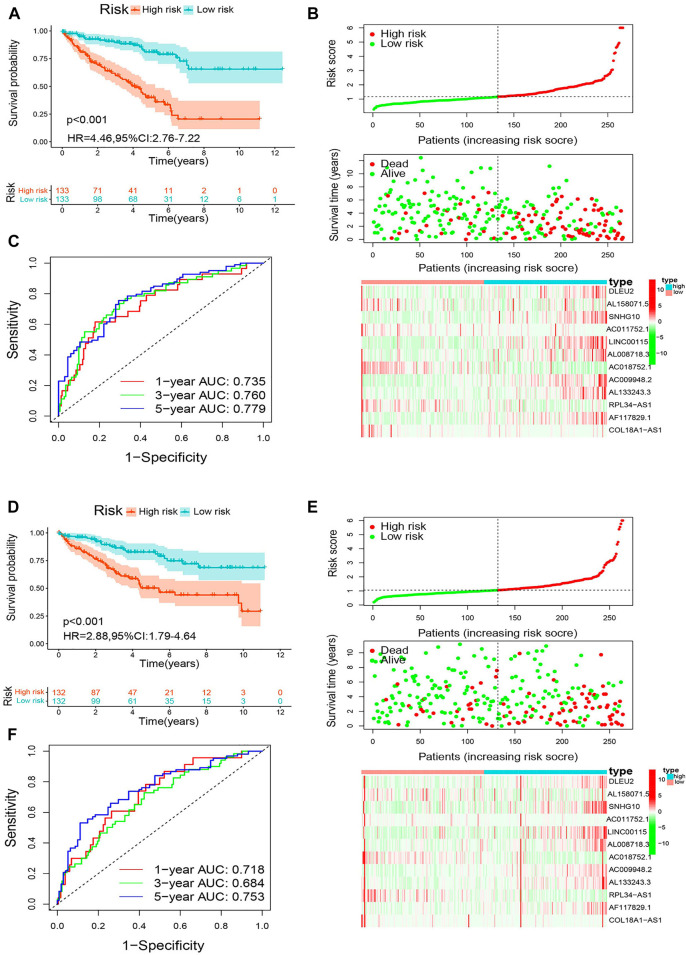
Analysis of the m6A-RLPS efficacy in patients stratified by risk level. **(A)** Kaplan–Meier curves of overall survival (OS) for the m6A-RLPS **(B)** Distributions of risk scores, survival status, and relative lncRNA expression; and **(C)** ROC curves for predicting 1-, 3-, and 5-years OS rates in the training cohort. (D) Kaplan–Meier curves of OS for the m6A-RLPS (E) Distributions of risk scores, survival status, and relative lncRNA expression; and (F) ROC curves for predicting 1-, 3-, and 5-years OS rates in the first validation cohort.

Next, the patients were classified into high- and low-risk groups based on the median m6A-RLPS risk score. Kaplan–Meier curves showed that in the training cohort, patients in the low-risk group had an improved OS compared to those in the high-risk group (*p* < 0.001, Figure 7A). As shown in Figure 7B, in the training cohort, the low-risk groups had an obviously higher survival rate and lower values for the risk score. Moreover, as the risk score increased, the expression of the protective lncRNAs (AL158071.5, AC011752.1, AC018752.1, RPL34-AS1, and COL18A1-AS1) decreased, whereas those of the risk lncRNAs (DLEU2, SNHG10, LINC00115, AL008718.3, AC009948.2, AL133243.3, and AF117829.1) increased. The ROC curves showed that the m6A-RLPS was accurate in predicting OS in the training cohort, and the AUCs for the 1-, 3-, and 5-years OS rates were 0.735, 0.760, and 0.779, respectively (Figure 7C). Similar findings were obtained in the first (Figures 7D–F), second (Supplementary Figure S4A–C), and third (Supplementary Figure S4D–F) validation cohorts. In particular, the efficacy of m6A-RLPS for predicting DSS in all the cohorts was also satisfactory (Supplementary Figure S5). These findings indicated that the prognostic signature has a robust and stable predictive efficacy.

### The m6A-Related lncRNA Prognostic Signature Is an Independent Prognostic Indicator in Clear-Cell Renal Cell Carcinoma

**FIGURE 8:**
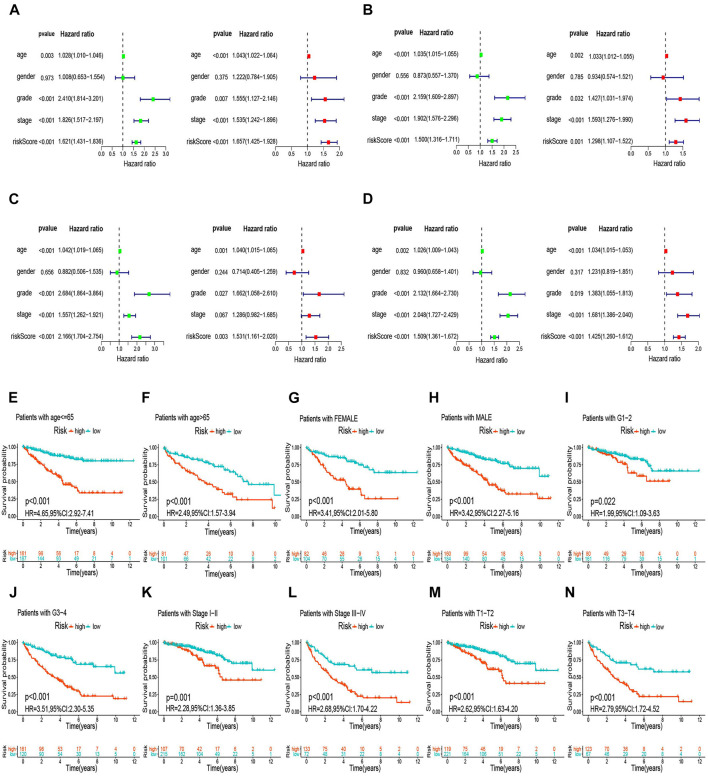
The m6A-RLPS is an independent prognostic indicator for overall survival (OS). Univariable and multivariate Cox regression analyses in the training **(A)**, first **(B)**, second **(C)**, and third **(D)** validation cohorts. Analysis of OS stratified by age **(E, F)**, sex **(G, H)**, tumor grade **(I, J)**, clinical stage, **(K, L)**, and T stage **(M, N)**.

We used univariable analysis to evaluate the independent prognostic significance of the m6A-RLPS risk score and several clinical features, including age, sex, tumor grade, and tumor stage (TNM stage was excluded due to incomplete information), in ccRCC. Age, tumor grade, tumor stage, and risk score were closely associated with OS and DSS, and multivariate analysis validated that these four variables were independent prognostic factors in patients with ccRCC (Figures 8A–D, Supplementary Figure S6A–D). Tests for interaction revealed no significant association between the effect of risk score and clinicopathological factors on the OS and DSS (all *p* values for interactions > 0.05) (Supplementary Table S4). Stratification survival analysis to estimate the predictive ability of m6A-RLPS in ccRCC patients with different clinicopathology revealed that high-risk patients had a worse OS and DSS than low-risk patients in every subgroup (Figure 8E–N, Supplementary Figure S6E-N), indicating the good predictive performance of the m6A-RLPS.

A nomogram for the 3- and 5-years OS rates based on the independent predictors determined using the multivariate analysis are shown in Supplementary Figure S7A. A point was plotted for each covariate, and a total nomogram score correlated with the 3- and 5-years OS rates was calculated for each patient. The nomogram showed a favorable accuracy in predicting OS, with a C-index of 0.80 (95% CI: 0.76–0.84), 0.76 (95% CI: 0.70–0.82), 0.78 (95% CI: 0.72–0.84), and 0.78 (95% CI: 0.74–0.82) for the training, first, second, and third validation cohorts, respectively. Moreover, calibration curves revealed that there was an appreciable agreement between the predictive outcome and actual survival, and similar findings were obtained in the validation cohorts (Supplementary Figure S7B–I).

### The m6A-Related lncRNA Prognostic Signature Correlates With Clinicopathology and TME Immune Activity

**FIGURE 9:**
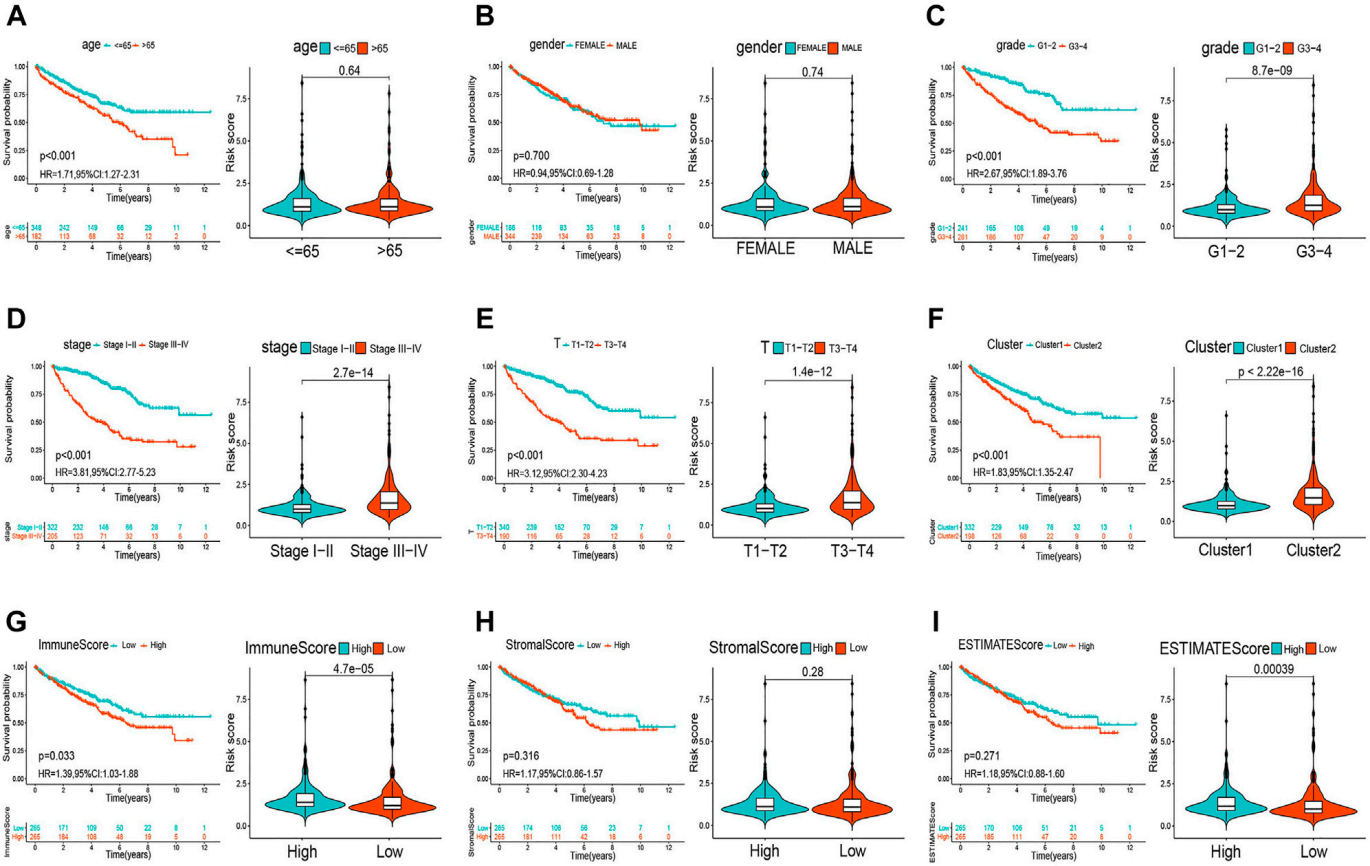
Relationships between the risk score and clinicopathological parameters. Distribution of risk scores according to age **(A)**, sex **(B)**, tumor grade **(C)**, clinical stage **(D)**, T stage **(E)**, cluster **(F)**, immune score **(G)**, stromal score **(H)**, and ESTIMATE score **(I)**.

The heatmap in Supplementary Figure S8 shows that the expression of the lncRNAs included in the m6A-RLPS was significantly correlated with cluster type, immune score, tumor grade, tumor stage, and T stage (all *p* < 0.01). In addition, a Student *t*-test revealed that the risk score increased with increasing tumor grade, clinical stage, T stage, immune score, and ESTIMATE score (Figures 9A–E, G–I), and that high-risk patients tended to be classified into cluster 2 (Figure 9F). These findings suggested that the lncRNAs included in the m6A-RLPS can influence the progression, malignancy, and survival outcomes of ccRCC.

**FIGURE 10:**
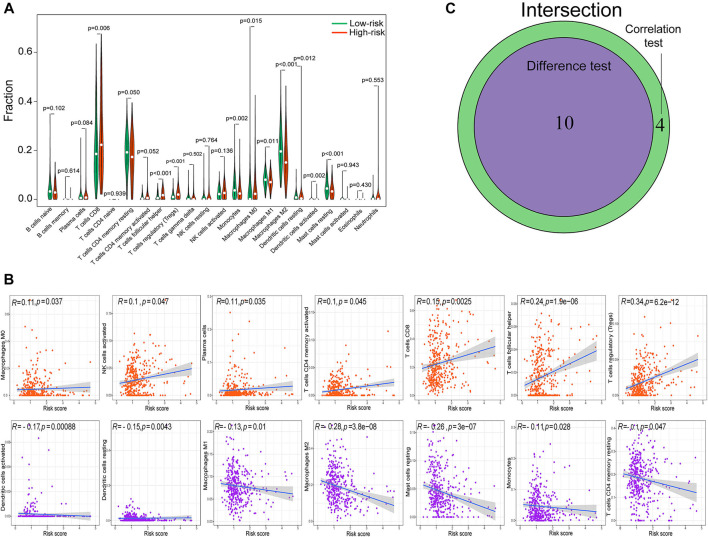
The m6A-RLPS is significantly associated with tumor-infiltrating immune cells (TICs). **(A)** Violin plots of the difference analysis confirming 10 types of TICs, including activated dendritic cells, resting dendritic cells, resting mast cells, M0, M1, and M2 macrophages, monocytes, CD8 T cells, follicular helper T cells, and Tregs, all *p* < 0.05. **(B)** Correlation analysis determined 14 types of TICs. **(C)** Venn diagram of common TICs.

Given the strong association between clinicopathology and TME immune activity, we comprehensively investigated the correlation between the m6A-RLPS risk score and immune cell infiltration. By combining difference and correlation analyses, we found that 10 types of TICs, including activated dendritic cells, resting dendritic cells, resting mast cells, M0, M1, and M2 macrophages, monocytes, CD8 T cells, follicular helper T cells, and regulatory T cells (Tregs), were strikingly associated with the m6A-RLPS (Figure 10). Of these cell types, four were positively correlated with the m6A-RLPS (M0 macrophages, CD8 T cells, follicular helper T cells, and Tregs), whereas the others were negative correlated.

**FIGURE 11:**
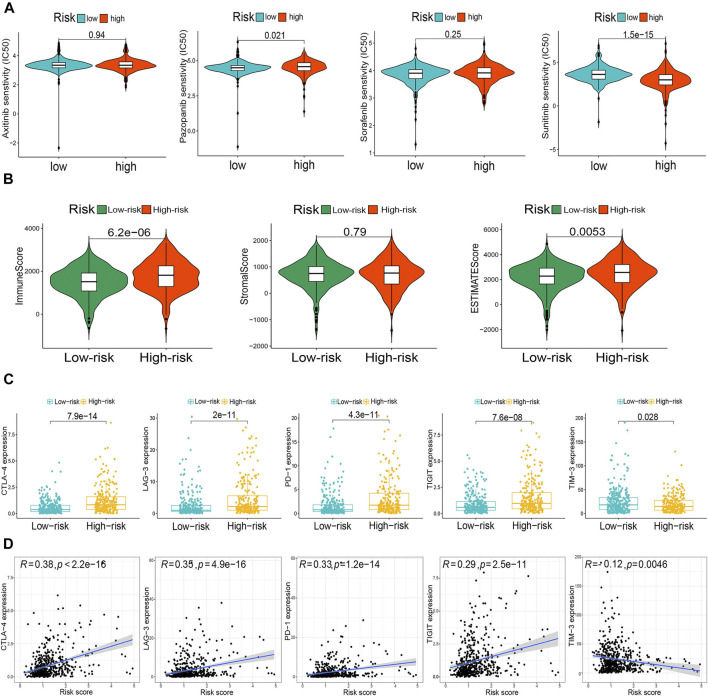
The m6A-RLPS is related with the tumor microenvironment (TME) immune reaction. **(A)** Differential drug sensitivity. **(B)** Distribution of TME scores in the risk groups. **(C)** Differential expression of five immune checkpoints in the risk groups. **(D)** Nearly all selected immune checkpoints correlated positively with the risk score.

Finally, we compared drug sensitivity to some TKIs, including axitinib, pazopanib, sorafenib, and sunitinib, between the risk groups. The risk stratification was significantly associated with pazopanib and sunitinib sensitivity (Figure 11A). In addition, the m6A-RLPS had a strong positive correlation with the TME scores obtained using the ESTIMATE algorithm (Figure 11B). Given the significant correlations of the routine immune checkpoints with the clusters, we next explored the associations between the m6A-RLPS and these immune checkpoints. The expression levels of all immune checkpoints, except TIM-3, were increased in high-risk patients and positively correlated with the risk score, reflecting the effect of the immune checkpoints on the TME and poor oncological outcomes (Figures 11C,D).

## Discussion

Due to the complexity and heterogeneity of ccRCC, patients with ccRCC generally have a high risk of recurrence and metastasis, as well as a dismal prognosis (Shingarev and Jaimes, 2017). Because of the limited predictive capability of traditional prognostic models, an m6A-related lncRNA-based prognostic model may improve the understanding and management of ccRCC (Veeratterapillay et al., 2012). Therefore, in this study, by systematically screening and identifying target lncRNAs, we created a reliable prognostic model with a satisfactory predictive capability for ccRCC outcomes.

We conducted a comprehensive ccRCC RNA-seq analysis using data of 539 patients with ccRCC downloaded from TCGA database. In total, 239 m6A-RLs were filtered out, 44 of which were determined to have prognostic value. Next, we divided all ccRCC samples into two clusters by consensus clustering to shed light on the potential effects of the m6A-RLs. The cluster subtypes were strongly linked to the OS of the patients with ccRCC, and cluster 2, comprising patients with worse prognosis, was associated with malignancy-related signaling pathways and numerous immune-related activities as indicated by GSEA. Further analyses emphasized the close relationships among the m6A-RLs, TICs, and immune checkpoints, which indicated that the expression pattern of m6A-RLs is remarkably associated with immunity and oncogenesis. Twelve lncRNAs of the forty-four m6A-RLs were used to construct an m6A-RLPS, which stratified the patients with ccRCC into low- and high-risk groups and exhibited a substantial prognostic prediction performance. The expression of four lncRNAs (COL18A1-AS1, DLEU2, LINC00115, and SNHG10) of the 12 m6A-RLs in ccRCC and normal tissues was evaluated using the GEO data. Multivariate Cox regression analysis showed that the m6A-RLPS was an independent prognostic factor for OS and DSS, and the prognostic predictive capacity of the signature was validated in patients stratified according to clinicopathological parameters. By integrating the m6A-RLPS, age, and tumor grade and stage, we constructed a quantitative nomogram, which was highly accurate and reliable in estimating patient survival. In fact, previous studies have also shown some novel prognostic signatures of ccRCC. Zeng et al. (2019) constructed a prognostic model based on six-lncRNAs, and Chen et al. (2020) identified an m6A RNA methylation regulator-based signature, which were used for predicting the OS of ccRCC and showed a good prediction efficiency, consistent with our model. However, each of the previous studies mainly focused on differentially expressed lncRNAs or m6A regulators; they did not combine and explore the association between m6A modification and lncRNAs, and did not validate the efficacy of signature in predicting DSS of ccRCC.

Numerous studies have highlighted the fact that m6A modification in specific lncRNAs affects tumorigenesis and metastasis in cancer through various mechanisms; however, how it affects particular lncRNAs in ccRCC remains unclear. In hepatocellular carcinoma, METTL3-mediated m6A modification stabilizes the lncRNA LINC00958 transcript to increase the levels of hepatoma-derived growth factor, thus facilitating tumor growth (Zuo et al., 2020). YTHDF3 inhibits colorectal cancer progression by negatively modulating the lncRNA GAS5 to generate a GAS5-YAP-YTHDF3 negative feedback loop (Ni et al., 2019). Furthermore, lncRNAs can interact with m6A regulators to regulate their functions. For example, the lncRNA GAS5-AS1 enhances the stability of GAS5 by interacting with ALKBH5 to suppress the proliferation of cervical cancer (Wang et al., 2019). We found METTL3 to be a risk factor associated with poor prognosis of ccRCC, which was consistent with findings in one previous study, but in contrast to those in another study (Li et al., 2017; Chen et al., 2020); thus, the role of MELLT3 in ccRCC remains uncertain. Moreover, studies have revealed that METTL3 and RBM15/15B can modulate m6A modification of lncRNA-XIST, which has been shown to facilitate ccRCC tumorigenicity via the miR-302c/SDC1 axis (Patil et al., 2016; Zhang et al., 2017). Together, the findings suggest that interactions between m6A modification and lncRNAs play vital roles in cancer development and improve our understanding of ccRCC and may aid in identifying potential prognostic markers for this disease.

Among the 12 prognostic m6A modification-related lncRNAs included in the m6A-RLPS, DLEU2 has been associated with the prognosis of multiple types of cancers (Ghafouri-Fard et al., 2021). Chen et al. (2017) reported that DLEU2 downregulates miR-30a-5p, resulting in poor prognosis in patients with ccRCC. LINC00115 can target miR-489-3p through the PI3K/AKT/mTOR pathway to promote the progression of colorectal cancer (Feng et al., 2020), and it can be activated by TGF-β to regulate the tumorigenicity of glioblastoma (Tang et al., 2019). Zhu et al. (2020) reported that lncRNA SNHG10 contributes to the proliferation and invasion of osteosarcoma by activating Wnt/β-catenin signaling via sponging miR-182-5p. Yuan et al. (2020) suggested that SNHG10 accelerates gastric cancer cell proliferation and migration via targeting the miR-495-3p/CTNNB1 axis. Unfortunately, to date, only few studies have been conducted in ccRCC, and the remaining nine lncRNAs have been rarely studied. However, our results corroborate the prognostic value of these m6A-targeted lncRNAs and offer insights into their potential roles in ccRCC oncogenesis and progression.

There is increasing evidence for the significance of m6A modification and lncRNAs in the regulation of cancer immunity, including immune cell infiltration in the TME, and immune resistance and activation (Denaro et al., 2019; Gu et al., 2021). The TME plays an important role in the tumorigenesis and progression of ccRCC (Hinshaw and Shevde, 2019). Recently, ICIs combined with TKIs have been recommended as a first-line treatment for metastatic RCC and the TME is actively involved in the therapeutic efficacy of these treatments (Simonaggio et al., 2021). Our findings indicated that the m6A-RLs play vital roles in ccRCC immune status. Therefore, we thoroughly analyzed the association between the m6A-RLPS and TME immune activity. The results showed that the risk score was strongly related with the TME score. Among TICs, the levels of M0 macrophages, CD8 T cells, follicular helper T cells, and Tregs notably differed between the high- and low-risk groups and were positively correlated with the risk score. Notable, these TICs reportedly are associated with tumorigenesis, progression, and immunotherapy efficacy in ccRCC (Santagata et al., 2017; Kadomoto et al., 2019; Xiong et al., 2020). Recent studies focusing on immune checkpoints, such as CTLA-4, LAG-3, PD-1, TIGHT, and TIM-3, have uncovered that these can significantly regulate the cancer immune function of TICs, leading to the inhibition of immune surveillance (Carosella et al., 2015; Manieri et al., 2017; Andrews et al., 2019). Therefore, immunotherapy has emerged as a promising cancer therapeutic option, and the clinical utility of combinations of ICIs and TKIs in metastatic ccRCC is being extensively researched (Motzer et al., 2019; Powles et al., 2020). In our study, the risk subgroups were notably related to the sensitivity to pazopanib and sunitinib. More importantly, the expression levels of nearly all the above immune checkpoints were positively correlated with the risk score calculated with the prognostic signature, suggesting that an immunosuppressive microenvironment is causally related with poor prognosis, and indicating the potential utility of our m6A-RLPS in estimating the response to ICIs.

We employed and analyzed clinical and survival data from a large cohort of patients with ccRCC from the TCGA database and validated the differential expression of several lncRNAs between normal and tumor tissues based on the GEO data. Nonetheless, this study had some limitations. First, because of a lack of ccRCC samples and large independent clinical data, we were not able to clinically validate the findings. In addition, the detailed functional roles of the m6A-related lncRNAs in ccRCC remain to be identified.

In conclusion, we identified 12 m6A-related lncRNAs with a potential prognostic value and developed a prognostic and predictive m6A-RLPS, which may be useful in the investigation of the functional and molecular mechanisms involved in ccRCC oncogenesis and in the determination of treatment strategies and efficacy in patients with ccRCC.

## Data Availability

Publicly available datasets were analyzed in this study. These data can be found here: The Cancer Genome Atlas (https://portal.gdc.cancer. gov/), Gene Expression Omnibus (https://www.ncbi.nlm.nih.gov/geo/), and Lnc2Cancer 3.0 (http://bio-bigdata.hrbmu.edu.cn/lnc2cancer).

## Abbreviations

AUC, area under curve; ccRCC, clear-cell renal cell carcinoma; C-index, concordance index; DSS, disease-specific survival; GSEA, gene set enrichment analysis; ICI, immune-checkpoint inhibitor; lncRNA, long non-coding RNA; m6A-RL, m6A-related lncRNA; m6A-RLPS, m6A-related lncRNA prognostic signature; OS, overall survival; RNA-seq, RNA-sequencing; ROC, receiver operating-characteristic; TIC, tumor-infiltrating immune cell; TME, tumor microenvironment; TKI, tyrosine kinase inhibitor.
