# Supplementary figures and images for: N6-Methyladenosine-Related Long Non-coding RNA Signature Associated With Prognosis and Immunotherapeutic Efficacy of Clear-Cell Renal Cell Carcinoma
Source: Front Genet. 2021 Oct 15;12:726369. doi: 10.3389/fgene.2021.726369 (PMC8554127; doi:10.3389/fgene.2021.726369)

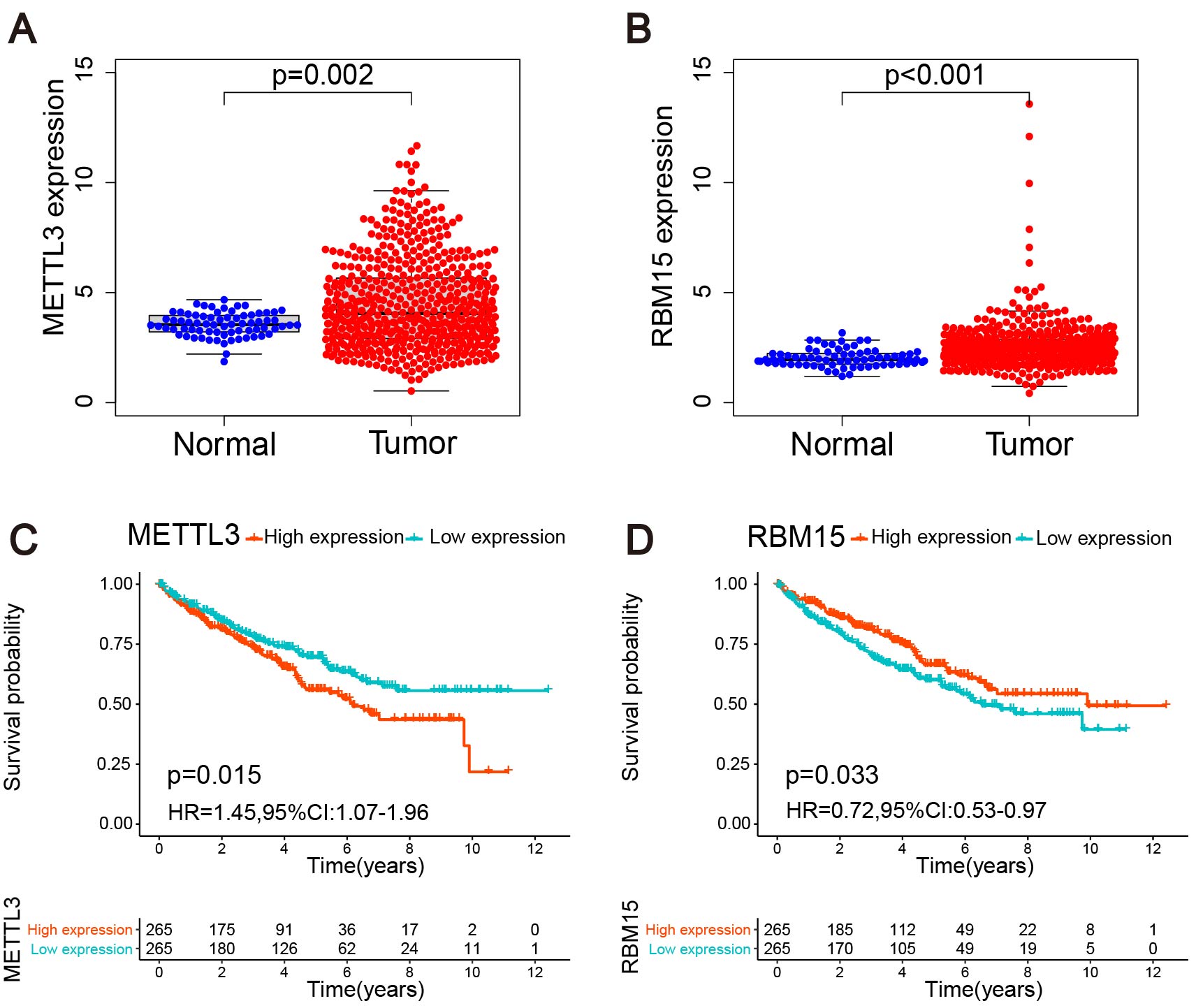

Supplement: Supplementary file 2 [file Image3.JPEG]

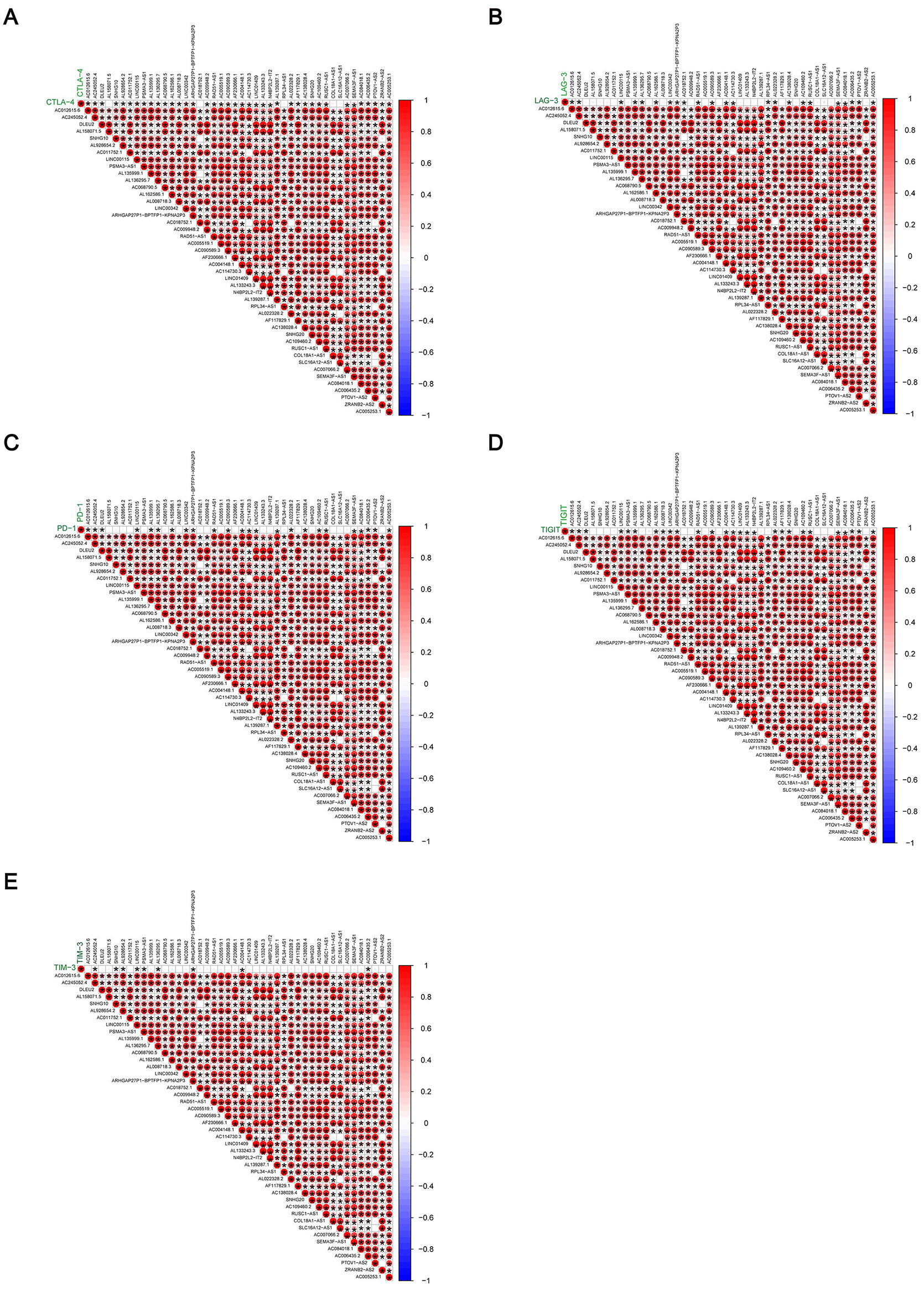

Supplement: Supplementary file 4 [file Image1.JPEG]

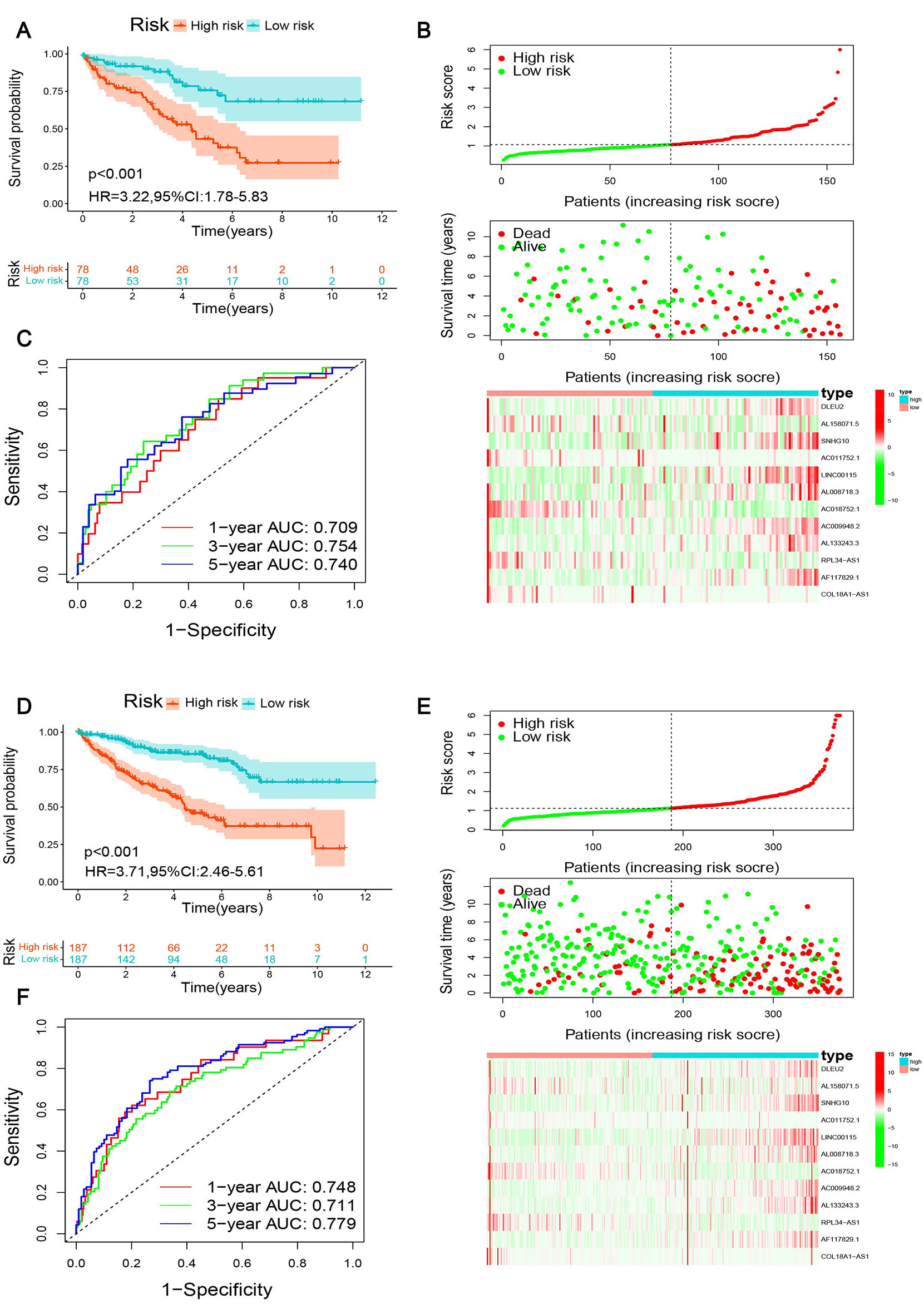

Supplement: Supplementary file 5 [file Image4.JPEG]

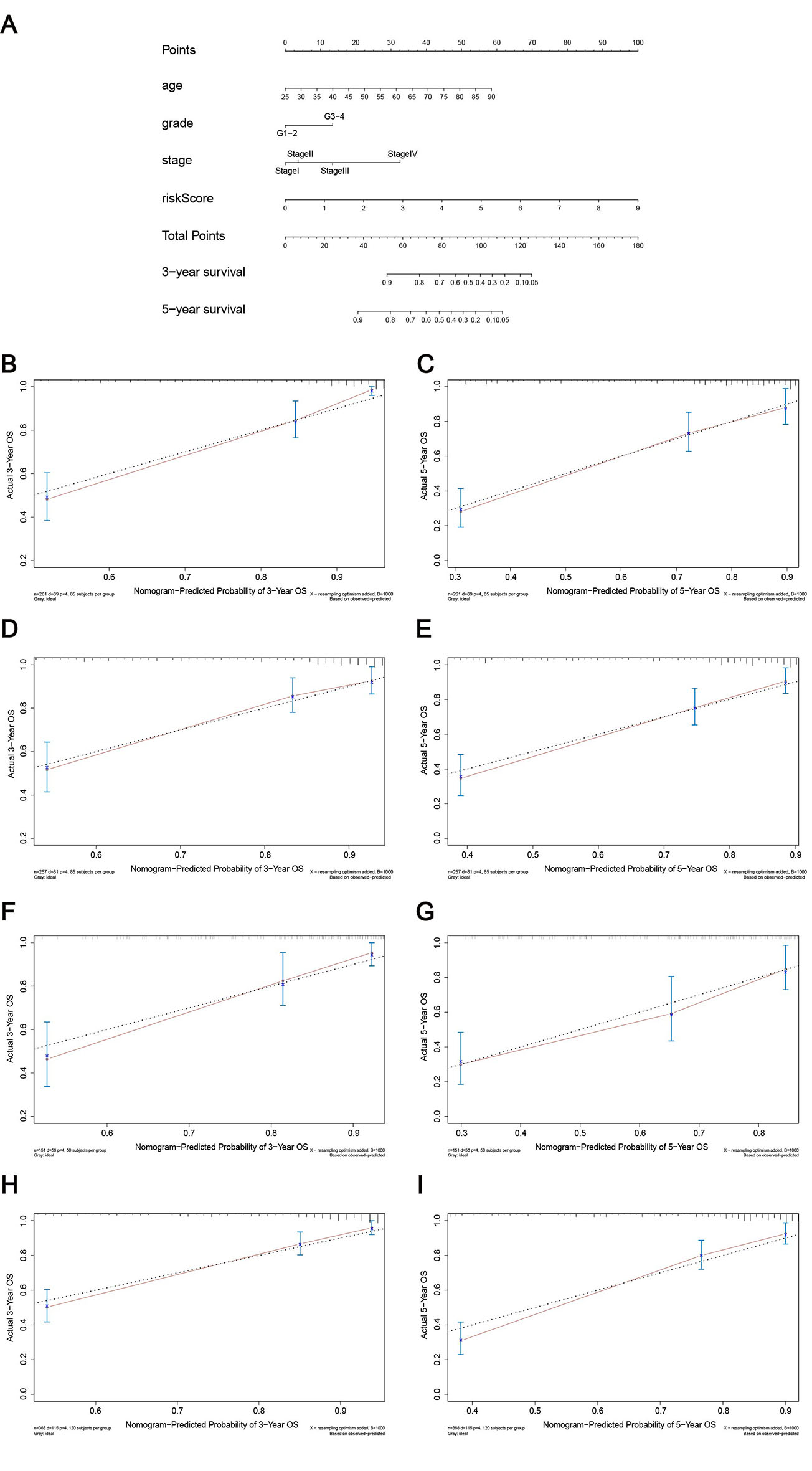

Supplement: Supplementary file 6 [file Image7.JPEG]

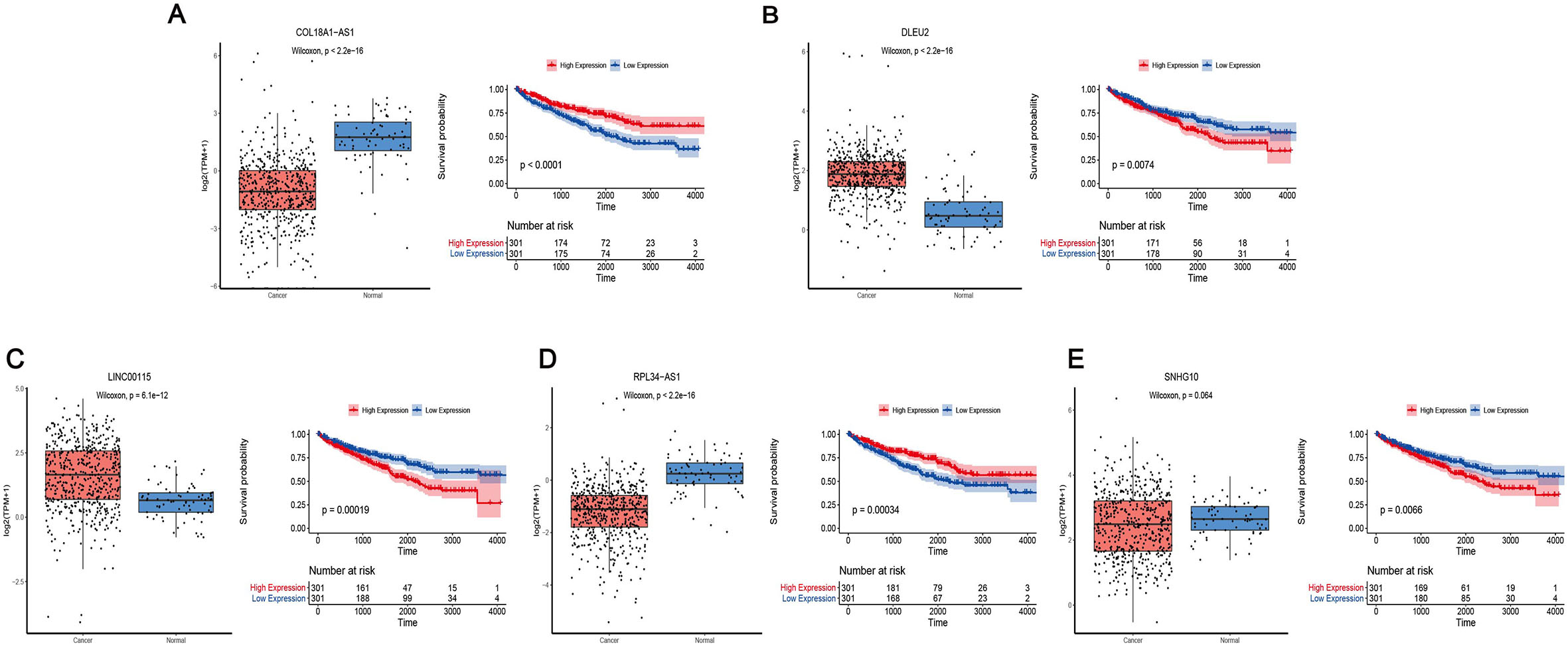

Supplement: Supplementary file 7 [file Image2.JPEG]

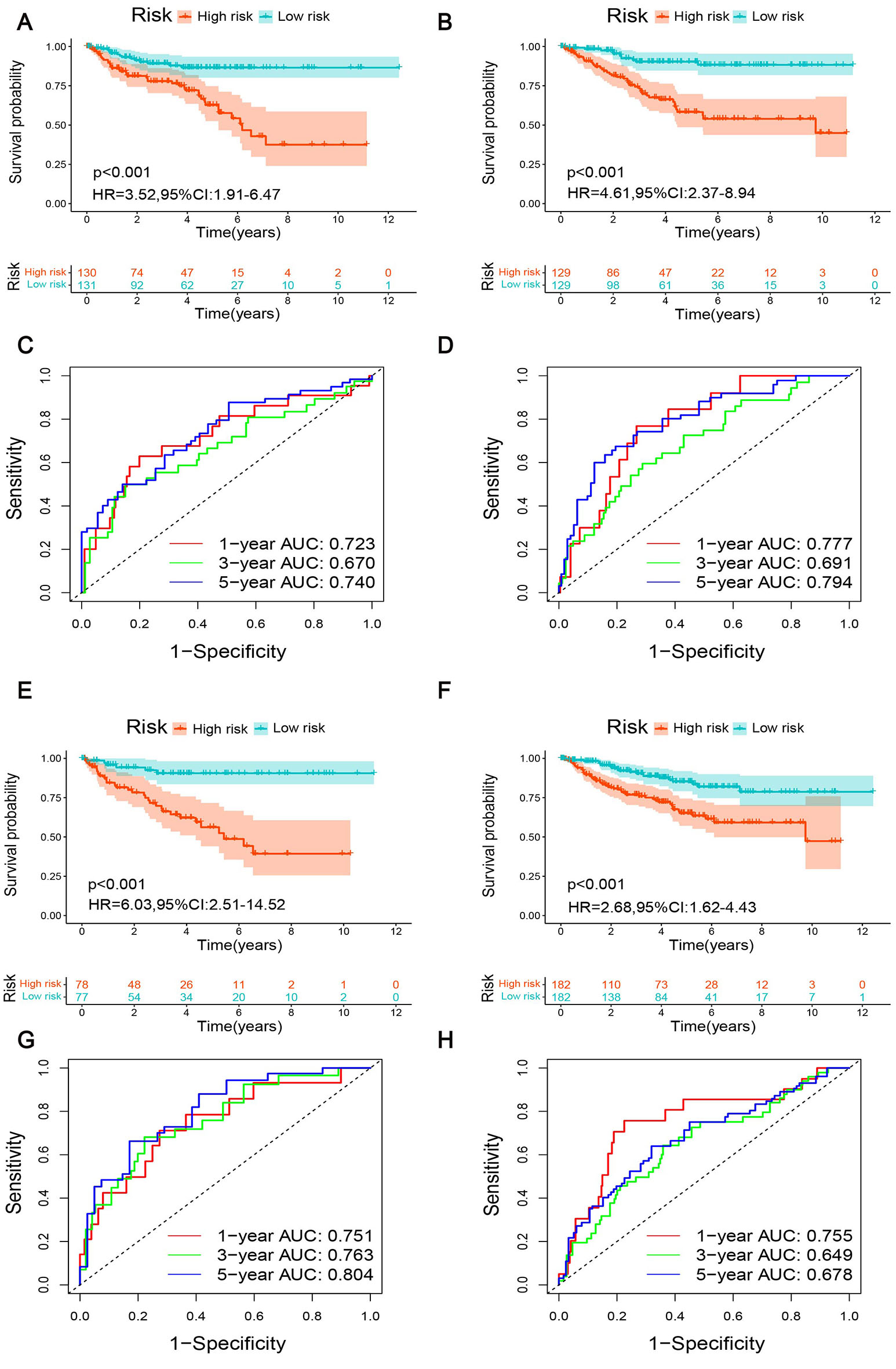

Supplement: Supplementary file 8 [file Image5.JPEG]

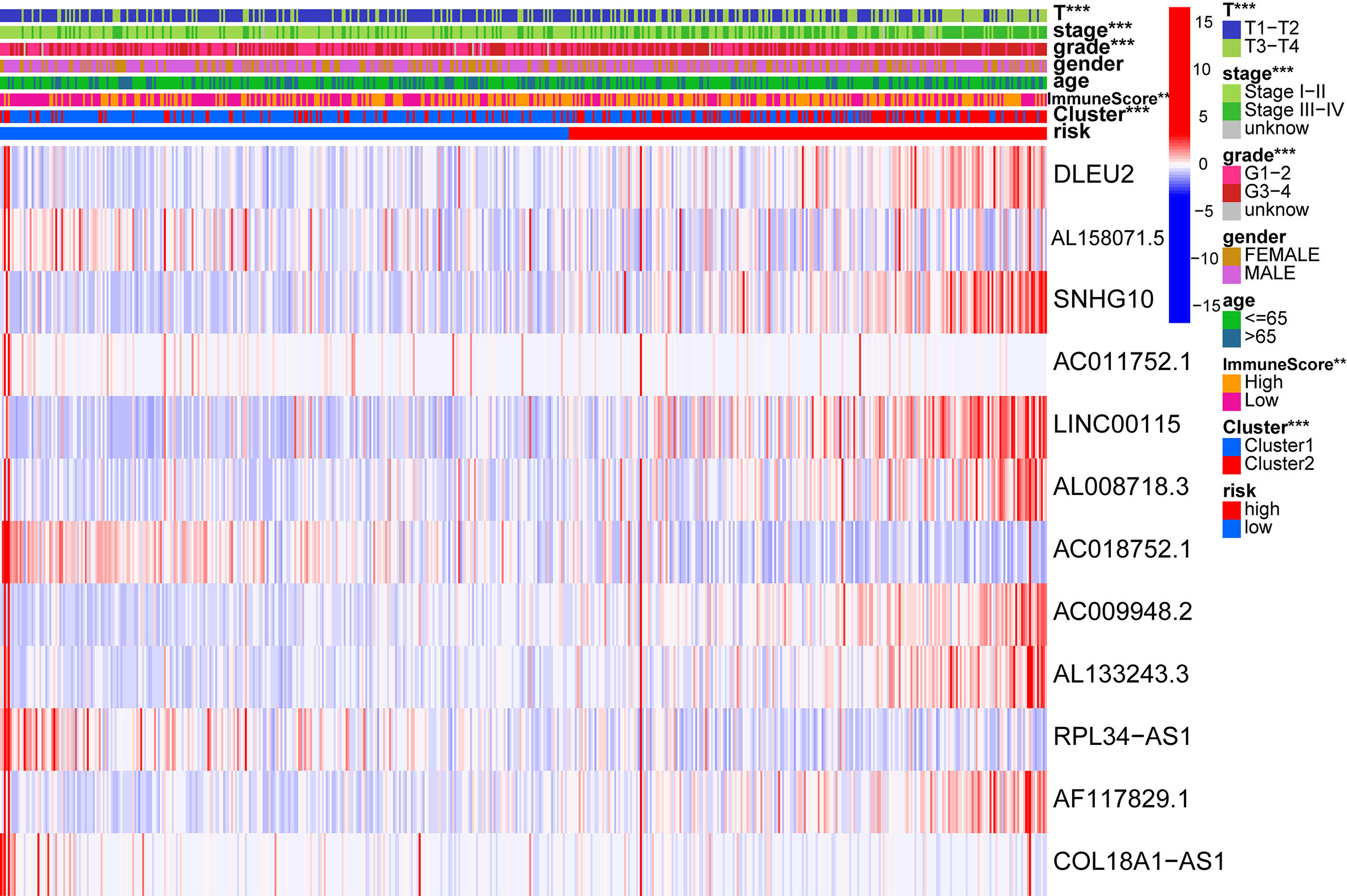

Supplement: Supplementary file 12 [file Image8.JPEG]

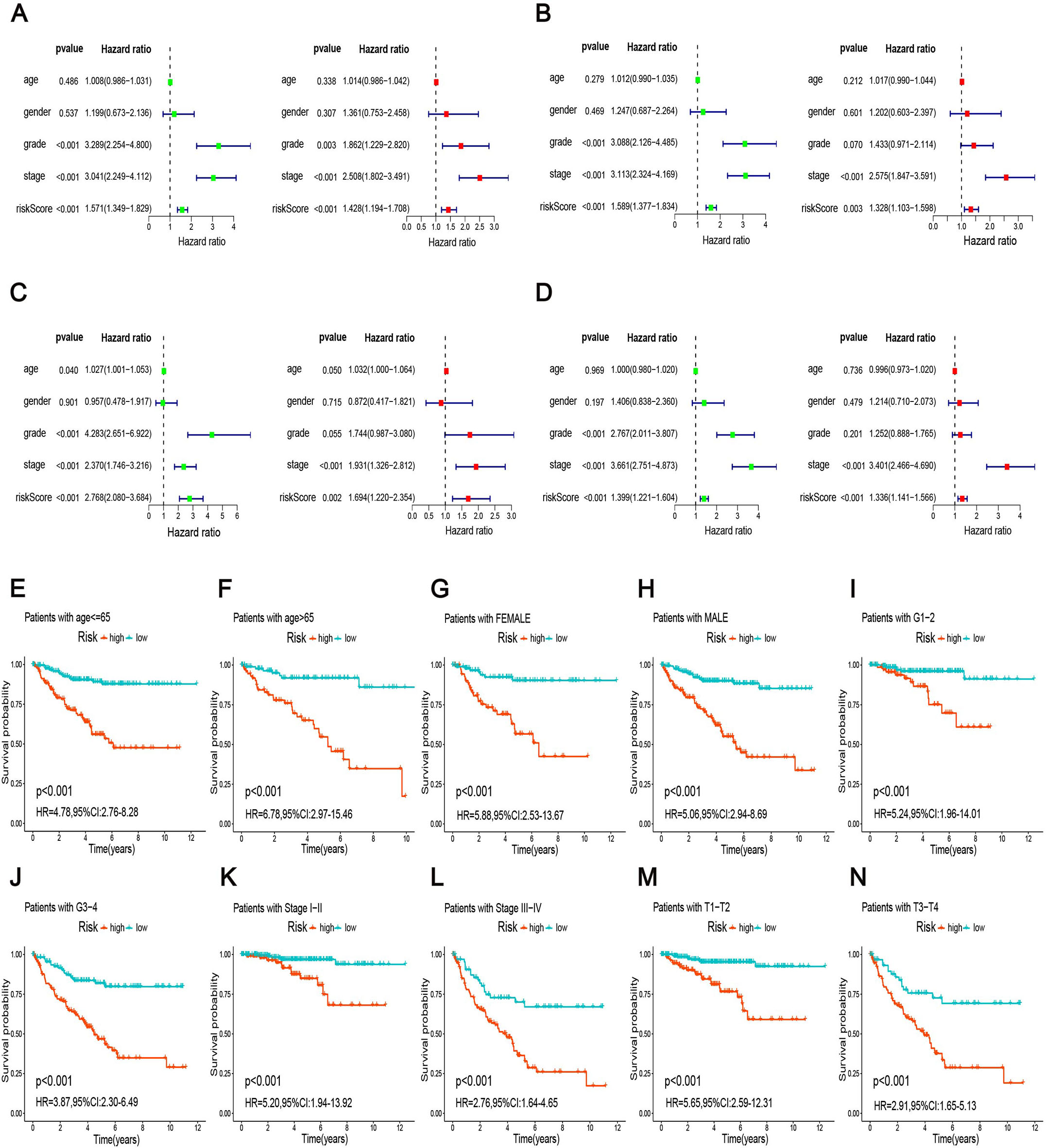

Supplement: Supplementary file 13 [file Image6.JPEG]
