# Supplementary material for: N6-Methyladenosine-Related Long Non-coding RNA Signature Associated With Prognosis and Immunotherapeutic Efficacy of Clear-Cell Renal Cell Carcinoma
Source: Front Genet. 2021 Oct 15;12:726369. doi: 10.3389/fgene.2021.726369 (PMC8554127; doi:10.3389/fgene.2021.726369)
Supplement: Supplementary file 3 [file Table1.DOCX]

**Table S1. Demographic and clinicopathological characteristics of patients with clear cell renal carcinoma(n=537).**

| **Characteristics** | **No. (%)** |
| --- | --- |
| **Age(years)** |  |
| ≤65 | 352(65.5%) |
| >65 | 185(34.5%) |
| **Gender** |  |
| Female | 191(35.6%) |
| Male | 346 (64.4%) |
| **Grade** |  |
| G1-2 | 244(45.4%) |
| G3-4 | 285(53.1%) |
| Unknown | 8(1.5%) |
| **Stage** |  |
| Stage I-II | 326(60.7%) |
| Stage III-IV | 208(38.7%) |
| Unknown | 3(0.6%) |
| **T stage** |  |
| T1-T2 | 344(64.1%) |
| T3-T4 | 193(35.9%) |
| Unknown | 0(0.0%) |
| **N stage** |  |
| N0 | 240(44.7%) |
| N1 | 17(3.2%) |
| Unknown | 280(52.1%) |
| **M stage** |  |
| M0 | 426(79.3%) |
| M1 | 79(14.7%) |
| Unknown | 32(6.0%) |
