# Supplementary material for: N6-Methyladenosine-Related Long Non-coding RNA Signature Associated With Prognosis and Immunotherapeutic Efficacy of Clear-Cell Renal Cell Carcinoma
Source: Front Genet. 2021 Oct 15;12:726369. doi: 10.3389/fgene.2021.726369 (PMC8554127; doi:10.3389/fgene.2021.726369)
Supplement: Supplementary file 9 [file Table3.DOCX]

**Table S3. The 44 m6A-related prognostic lncRNAs.**

| **m6A-related lncRNA** | **HR** | **HR.95L** | **HR.95H** | **P-value** |
| --- | --- | --- | --- | --- |
| **RPL34-AS1** | 0.0004 | 0.0000 | 0.0200 | 7.28E-05 |
| **ZRANB2-AS2** | 0.0065 | 0.0002 | 0.1994 | 3.92E-03 |
| **COL18A1-AS1** | 0.0426 | 0.0071 | 0.2579 | 5.90E-04 |
| **AC011752.1** | 0.0602 | 0.0083 | 0.4377 | 5.50E-03 |
| **AL158071.5** | 0.0615 | 0.0093 | 0.4085 | 3.89E-03 |
| **SLC16A12-AS1** | 0.4668 | 0.2716 | 0.8025 | 5.85E-03 |
| **AC018752.1** | 0.6150 | 0.5077 | 0.7450 | 6.73E-07 |
| **AL139287.1** | 1.0455 | 1.0109 | 1.0813 | 9.60E-03 |
| **PSMA3-AS1** | 1.0828 | 1.0260 | 1.1427 | 3.83E-03 |
| **RAD51-AS1** | 1.1277 | 1.0559 | 1.2043 | 3.42E-04 |
| **AL022328.2** | 1.1283 | 1.0402 | 1.2239 | 3.61E-03 |
| **AL928654.2** | 1.1354 | 1.0615 | 1.2145 | 2.19E-04 |
| **AC084018.1** | 1.1356 | 1.0740 | 1.2008 | 7.89E-06 |
| **PTOV1-AS2** | 1.1364 | 1.0817 | 1.1939 | 3.72E-07 |
| **SNHG20** | 1.1577 | 1.0585 | 1.2662 | 1.36E-03 |
| **LINC00342** | 1.1680 | 1.1110 | 1.2279 | 1.17E-09 |
| **AC138028.4** | 1.1928 | 1.0555 | 1.3479 | 4.71E-03 |
| **N4BP2L2-IT2** | 1.2533 | 1.0926 | 1.4377 | 1.26E-03 |
| **AC004148.1** | 1.2571 | 1.1550 | 1.3683 | 1.19E-07 |
| **AL136295.7** | 1.2691 | 1.1412 | 1.4114 | 1.10E-05 |
| **ARHGAP27P1-BPTFP1-KPNA2P3** | 1.2705 | 1.1327 | 1.4251 | 4.38E-05 |
| **AL162586.1** | 1.2769 | 1.1609 | 1.4046 | 4.98E-07 |
| **AC068790.5** | 1.3153 | 1.0976 | 1.5763 | 2.99E-03 |
| **AC109460.2** | 1.3185 | 1.0690 | 1.6263 | 9.78E-03 |
| **RUSC1-AS1** | 1.3238 | 1.1762 | 1.4898 | 3.30E-06 |
| **AC090589.3** | 1.3477 | 1.1747 | 1.5462 | 2.07E-05 |
| **AC006435.2** | 1.3632 | 1.1922 | 1.5588 | 5.87E-06 |
| **AC005519.1** | 1.3673 | 1.1195 | 1.6698 | 2.16E-03 |
| **AF117829.1** | 1.3800 | 1.1592 | 1.6429 | 2.94E-04 |
| **SNHG10** | 1.3954 | 1.2434 | 1.5660 | 1.49E-08 |
| **DLEU2** | 1.4389 | 1.1444 | 1.8093 | 1.85E-03 |
| **AL135999.1** | 1.4558 | 1.2500 | 1.6954 | 1.37E-06 |
| **AC005253.1** | 1.4607 | 1.1746 | 1.8166 | 6.58E-04 |
| **AC012615.6** | 1.5116 | 1.2559 | 1.8194 | 1.25E-05 |
| **LINC00115** | 1.6575 | 1.3677 | 2.0087 | 2.56E-07 |
| **AC114730.3** | 1.7468 | 1.3380 | 2.2805 | 4.12E-05 |
| **SEMA3F-AS1** | 1.7740 | 1.2086 | 2.6041 | 3.42E-03 |
| **AC007066.2** | 1.8751 | 1.2669 | 2.7751 | 1.67E-03 |
| **AL133243.3** | 2.3200 | 1.5638 | 3.4417 | 2.89E-05 |
| **AL008718.3** | 2.3615 | 1.4761 | 3.7779 | 3.38E-04 |
| **AC245052.4** | 2.6976 | 1.5624 | 4.6576 | 3.69E-04 |
| **AC009948.2** | 2.8847 | 1.6231 | 5.1269 | 3.05E-04 |
| **AF230666.1** | 4.0885 | 1.6924 | 9.8768 | 1.75E-03 |
| **LINC01409** | 6.1860 | 2.2801 | 16.7823 | 3.45E-04 |

m6A, N6-methyladenosine; lncRNA, long non-coding RNA; HR, hazard ratio.
