# Supplementary material for: N6-Methyladenosine-Related Long Non-coding RNA Signature Associated With Prognosis and Immunotherapeutic Efficacy of Clear-Cell Renal Cell Carcinoma
Source: Front Genet. 2021 Oct 15;12:726369. doi: 10.3389/fgene.2021.726369 (PMC8554127; doi:10.3389/fgene.2021.726369)
Supplement: Supplementary file 10 [file DataSheet2.docx]

Supplementary Material

**Supplementary Tables**

**Table S1.** Demographic and clinicopathological characteristics of patients with clear-cell renal carcinoma (n = 537).

**Table S2.** The 239 m6A-related lncRNAs and their associated m6A genes.

**Table S3.** The 44 m6A-related prognostic lncRNAs.

**Table S4.** Interaction tests between the risk score and clinicopathological characteristics on the overall survival (OS) and disease-specific survival (DSS).

**Supplementary Figures**

**Figure S1.** Correlations between the selected immune checkpoints and the 44 m6A-related lncRNAs. **p* < 0.05.

**Figure S2.** Differential expression and ovreall survival(OS) of the 5 m6A-RLs in the Lnc2Cancer 3.0 web tool.

**Figure S3.** Differential expression and OS of two co-expression m6A genes.

**Figure S4.** (A) Kaplan–Meier curves of the overall survival (OS) for the m6A-RLPS; (B) Distributions of risk scores, survival status, and relative lncRNA expressions; and (C) ROC curves for predicting 1-, 3-, and 5-year OS rates in the 2^nd^ validation cohort. (D) Kaplan-Meier curves of OS for the m6A-RLPS; (E) Distributions of risk scores, survival status, and relative lncRNA expression; and (F) ROC curves for predicting 1-, 3-, and 5-year OS rates in the 3^rd^ validation cohort.

**Figure S5.** Analysis of the m6A-RLPS efficacy for disease-specific survival (DSS). (A,B) Kaplan–Meier curves and (C,D) ROC curves in the training and 1^st^ validation cohorts. (E,F) Kaplan–Meier curves and (G,H) ROC curves in the2^nd^ and 3^rd^ validation cohorts.

**Figure S6.** The m6A-RLPS is an independent prognostic indicator for disease-specific survival (DSS). Univariable and multivariate Cox regression analyses in the training (A), 1^st^ (B), 2^nd^ (C), and 3^rd^ (D) validation cohorts. Survival analysis of DSS stratified by age (E, F), sex (G, H), tumor grade (I, J), clinical stage, (K, L), and T stage (M, N).

**Figure S7.** (A) Nomogram based on age, tumor grade, clinical stage, and risk score. Calibration curves of the nomogram for predicting the probability of overall survival (OS) at 3- and 5- years in the training (B,C), 1^st^ (D,E), 2^nd^ (F,G), and 3^rd^ (H,I) validation cohorts (bootstrap method, 1,000 repetitions).

**Figure S8.** Heatmap based on the chi-square test or Fisher’s exact test, illustrating association between the m6A-RLPS-based risk level and clinicopathological parameters. ***p* < 0.01, ****p* < 0.001.
