# Supplementary material for: N6-Methyladenosine-Related Long Non-coding RNA Signature Associated With Prognosis and Immunotherapeutic Efficacy of Clear-Cell Renal Cell Carcinoma
Source: Front Genet. 2021 Oct 15;12:726369. doi: 10.3389/fgene.2021.726369 (PMC8554127; doi:10.3389/fgene.2021.726369)
Supplement: Supplementary file 11 [file Table4.DOCX]

**Table S4.** Interaction tests between the risk score and clinicopathological characteristics on the overall survival (OS) and disease-specific survival (DSS).

|  | OS (P value for interaction) | | | |  |  | DSS (P value for interaction) | | | |
| --- | --- | --- | --- | --- | --- | --- | --- | --- | --- | --- |
|  | training cohort | 1^st^ validation cohort | 2^nd^ validation cohort | 3^rd^ validation cohort |  |  | training cohort | 1^st^ validation cohort | 2^nd^ validation cohort | 3^rd^ validation cohort |
| age | 0.373 | 0.330 | 0.089 | 0.394 |  |  | 0.837 | 0.897 | 0.306 | 0.523 |
| gender | 0.202 | 0.124 | 0.987 | 0.230 |  |  | 0.299 | 0.104 | 0.313 | 0.712 |
| grade | 0.882 | 0.526 | 0.266 | 0.151 |  |  | 0.433 | 0.232 | 0.109 | 0.667 |
| stage | 0.873 | 0.886 | 0.425 | 0.873 |  |  | 0.065 | 0.762 | 0.264 | 0.253 |
